# Chloroquine Intervenes Nephrotoxicity of Nilotinib through Deubiquitinase USP13‐Mediated Stabilization of Bcl‐XL

**DOI:** 10.1002/advs.202302002

**Published:** 2023-07-14

**Authors:** Hao Yan, Xiangliang Huang, Jiangxin Xu, Ying Zhang, Jiajia Chen, Zhifei Xu, Hui Li, Zeng Wang, Xiaochun Yang, Bo Yang, Qiaojun He, Peihua Luo

**Affiliations:** ^1^ Center for Drug Safety Evaluation and Research of Zhejiang University College of Pharmaceutical Sciences Zhejiang University Hangzhou 310058 China; ^2^ Department of Pharmacy Zhejiang Cancer Hospital Hangzhou 310005 China; ^3^ Institute of Pharmacology & Toxicology College of Pharmaceutical Sciences Zhejiang University Hangzhou 310058 China; ^4^ Innovation Institute for Artificial Intelligence in Medicine of Zhejiang University Hangzhou 310018 China; ^5^ Department of Cardiology Second Affiliated Hospital School of Medicine Zhejiang University Hangzhou 310009 China

**Keywords:** Bcl‐XL, cell apoptosis, chloroquine, nephrotoxicity, nilotinib, ubiquitin specific peptidase 13 (USP13)

## Abstract

Nephrotoxicity has become prominent due to the increase in the clinical use of nilotinib, a second‐generation BCR‐ABL1 inhibitor in the first‐line treatment of Philadelphia chromosome‐positive chronic myeloid leukemia. To date, the mechanism of nilotinib nephrotoxicity is still unknown, leading to a lack of clinical intervention strategies. Here, it is found that nilotinib could induce glomerular atrophy, renal tubular degeneration, and kidney fibrosis in an animal model. Mechanistically, nilotinib induces intrinsic apoptosis by specifically reducing the level of BCL2 like 1 (Bcl‐XL) in both vascular endothelial cells and renal tubular epithelial cells, as well as in vivo. It is confirmed that chloroquine (CQ) intervenes with nilotinib‐induced apoptosis and improves mitochondrial integrity, reactive oxygen species accumulation, and DNA damage by reversing the decreased Bcl‐XL. The intervention effect is dependent on the alleviation of the nilotinib‐induced reduction in ubiquitin specific peptidase 13 (USP13) and does not rely on autophagy inhibition. Additionally, it is found that USP13 abrogates cell apoptosis by preventing excessive ubiquitin‒proteasome degradation of Bcl‐XL. In conclusion, the research reveals the molecular mechanism of nilotinib's nephrotoxicity, highlighting USP13 as an important regulator of Bcl‐XL stability in determining cell fate, and provides CQ analogs as a clinical intervention strategy for nilotinib's nephrotoxicity.

## Graphical abstract

The mechanism underlying nilotinib's nephrotoxicity during anticancer therapy is still elusive. Here, the transcription inhibition of ubiquitin specific peptidase 13 (USP13) by nilotinib is shown, resulting in the excessive degradation of Bcl‐XL, which contributes to cell apoptosis and the formation of nephrotoxicity. Chloroquine and its analog possess their therapeutic potential by regulating USP13 and do not rely on autophagy inhibition.

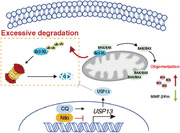

## 1 Introduction

Nilotinib is a second‐generation BCR‐ABL1 tyrosine kinase inhibitor that has been widely approved in many countries for the first‐line treatment of patients with newly diagnosed Philadelphia chromosome‐positive chronic myeloid leukemia (CML) in the chronic phase or imatinib‐resistant or imatinib‐intolerant CML in the chronic phase or accelerated phase.^[^ ^1^ ^]^ However, according to the results of clinical trials, ≈17% of patients receiving nilotinib have developed renal impairment, and the rates rise to 67% in patients with basal renal dysfunction, which extremely affects the quality life of patients.^[^ ^2^ ^]^ The underlying mechanism of nilotinib‐induced nephrotoxicity is still obscure, leading to a lack of effective interventions in clinics. Persistent renal complications demand dose reduction or discontinuation of the treatment, which will cause new problems, such as the progression of cancer. Thus, systematic investigation of the key molecular mechanism and searching for a precise innovative intervention strategy for nilotinib nephrotoxicity is of great significance to guide its safe clinical application.

Apoptosis has been recognized as a physiologic form of programmed cell death,^[^ ^3^ ^]^ which is closely associated with drug‐induced nephrotoxicity.^[^ ^4^ ^]^ Apoptosis is mostly initiated by the extrinsic signaling pathway or intrinsic signaling pathway,^[^ ^5^ ^]^ and the latter is extensively reported to be involved in drug‐induced cell death.^[^ ^6^ ^]^ The intrinsic pathway of apoptosis is orchestrated in a sophisticated manner by BCL2 apoptosis regulator (Bcl‐2) family members, which can be divided into proapoptotic proteins and antiapoptotic proteins based on their diverse roles in apoptosis.^[^ ^7^ ^]^ BCL2 associated X, apoptosis regulator (BAX) and BCL2 antagonist/killer 1 (BAK) are two of the most classic proapoptotic proteins.^[^ ^8^ ^]^ The activation of BAX and BAK in response to multiple stimuli will enable them to oligomerize and form macropores in the membrane, which causes mitochondrial outer membrane permeabilization and, finally, the induction of apoptosis.^[^ ^5^, ^9^ ^]^ Antiapoptotic proteins, including Bcl‐2 and BCL‐2 like 1 (Bcl‐XL), can bind to proapoptotic proteins and subsequently prevent the induction of apoptosis.^[^ ^5^, ^10^ ^]^ Relevant studies have shown that the balance between the intracellular content of proapoptotic proteins and antiapoptotic proteins determines cell fate. Suberoylanilide hydroxamic acid, a novel anticancer agent, was reported to decrease Bcl‐2 and Bcl‐XL and activate cell apoptosis in renal tubular cells.^[^ ^11^ ^]^ Xiong et al. also found that irbesartan could intervene in contrast media‐induced renal tubular epithelial cell damage by regulating the ratio of BAX and Bcl‐2.^[^ ^12^ ^]^ The intracellular content of the Bcl‐2 protein family was subjected to tight regulation in terms of transcription, translation and protein turnover (ubiquitin–proteasome pathway, autophagy, etc.), which was disturbed in the presence of harmful stimuli.^[^ ^7b^ ^]^ A previous study demonstrated that the ubiquitin‐dependent pathway is the classical pathway that regulates the Bcl‐2 protein family;^[^ ^13^ ^]^ however, whether such a regulatory pattern occurs in drug‐induced nephrotoxicity has rarely been reported.

Ubiquitination is a widespread posttranslational modification that regulates protein stability and involves different functional enzymes: activating (E1)‐conjugating (E2)‐ligating (E3) ubiquitin enzymes.^[^ ^14^ ^]^ Meanwhile, deubiquitinases (DUBs) can reversibly remove the attached ubiquitin chain from substrates to prevent ubiquitin‐mediated protein degradation.^[^ ^15^ ^]^ Ubiquitin specific peptidase 13 (USP13) is a DUB whose expression is correlated with the control of cell apoptosis.^[^ ^16^ ^]^ For instance, USP13 was able to indirectly mediate the protein stability of p53 by regulating its deubiquitinating protease USP10.^[^ ^17^ ^]^ Moreover, USP13 was reported to interact with and stabilize MCL1 apoptosis regulator, BCL2 family member (MCL‐1) by deubiquitinating enzymatic activity, hence inhibiting cell apoptosis.^[^ ^18^ ^]^ Considering the pivotal role of cell apoptosis in kidney diseases, it is reasonable to deduce the correlation between USP13 and nephrology. Xie et al. discovered that USP13 was essential for the progression of clear cell renal cell carcinoma, and this effect was mediated by the regulation of ZHX2.^[^ ^19^ ^]^ In addition, little is known about the relevance between USP13 and other kidney diseases.

Chloroquine (CQ) and its derivative hydroxychloroquine (HCQ) have been clinically used in the management of malaria and autoimmune diseases for long periods.^[^ ^20^ ^]^ In addition, CQ has been proven in multiple clinical trials to inhibit tumor drug resistance and metastasis, hence enhancing the efficacy of chemotherapeutic drugs.^[^ ^21^ ^]^ Meanwhile, CQ is a classic autophagy inhibitor that can prevent the fusion of autophagosomes and lysosomes to inhibit lysosomal function, which is widely used in the study of autophagy. Given the importance of CQ in the field of autophagy, researchers generally attribute the pharmacological mechanism of CQ to the inhibition of autophagy, with little attention given to its autophagy‐independent effects. In fact, several studies have confirmed that CQ functions independently of autophagy. Maes et al. found that CQ promoted tumor vessel normalization by upregulating the notch receptor 1 (NOTCH1) pathway, and Hirata et al. revealed that CQ inhibited glutamate‐induced death of a neuronal cell line by regulating sigma non‐opioid intracellular receptor 1 (SIGMAR1).^[^ ^22^ ^]^ This is a breakthrough in the study of the nonautophagy‐dependent effect of CQ, which broadens the application field of CQ. More effective targets need to be further explored.

In this study, we focused on the clinical dilemma of nilotinib‐induced renal adverse reactions and set out to unveil the mechanism of nilotinib's nephrotoxicity. Mechanistically, nilotinib downregulated the antiapoptotic protein Bcl‐XL in kidney cells, which ultimately activated the intrinsic pathway of apoptosis, a primary feature of kidney injury. In addition, we found that CQ, whose mechanism was independent of generally perceived autophagy inhibition, could significantly alleviate nilotinib‐induced kidney cell apoptosis by regulating the expression of USP13. Overall, our study was expected to clarify the molecular mechanism of nilotinib's nephrotoxicity, characterize a novel autophagy‐independent mechanism of CQ to intervene with nilotinib's nephrotoxicity and provide new strategies for clinical intervention.

## 2 Results

### 2.1 Nilotinib Induces Nephrotoxicity In Vivo

**Figure 1:**
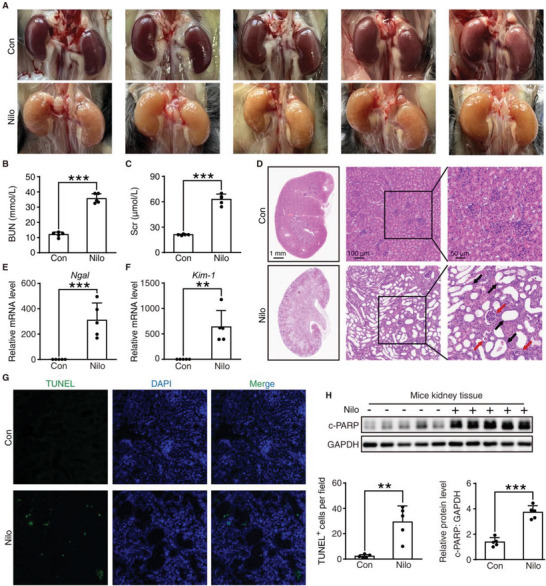
Nilotinib induces nephrotoxicity in vivo. A–H) C57BL/6J mice were administered 0.5% CMC‐Na or nilotinib (300 mg kg^−1^) by gavage for 30 days. Kidneys and serum were collected (*n* = 5 per group). A) Representative photos of kidneys from control group and nilotinib‐treated group. B) Blood urea nitrogen (BUN) and C) Serum creatinine (Scr) levels were analyzed. D) Representative panoramic images and local zoomed images of kidney tissues with hematoxylin and eosin staining (H&E), Scale bar = 1 mm, 100 µm, or 50 µm. Red arrows indicated the death of glomerular vascular endothelial cells and glomerular atrophy. Black arrows indicated the degeneration and expansion of renal tubular. E,F) The mRNA expression of *Ngal* and *Kim‐1* were analyzed by qRT‐PCR. G) Fluorescence microscope images of kidney tissues stained with TUNEL and DAPI. Scale bar = 50 µm. Quantitative analysis was performed to detect apoptotic cells. H) Relative expression of c‐PARP in kidney tissues was analyzed by Western blot with GAPDH as a loading control. The results are presented as the mean ± SD. The *P* value was calculated by Student's *t*‐test (unpaired, two‐tailed, two groups). **P < 0.01; ***P < 0.001.

To reproduce the nephrotoxicity of nilotinib, we treated C57BL/6J mice with CMC‐Na or nilotinib (300 mg kg^−1^) by gavage daily for 30 days. The dosage of nilotinib was calculated according to the Meen–Rubner equation and was three times the clinically recommended dose.^[^ ^23^ ^]^ After 1 month of nilotinib treatment, kidneys and serum were collected for further analysis. Compared to the control group, nilotinib treatment resulted in apparent abnormalities in kidney appearance (**Figure** **1**A) as well as elevated blood urea nitrogen (BUN) and serum creatinine (Scr) (Figure 1B,C), suggesting severe renal impairment in the presence of nilotinib. A clinical case report showed that one patient receiving nilotinib treatment developed serious renal dysfunction whose pathological phenotype was characterized by the shedding of vascular endothelial cells of the renal glomeruli and hyperplasia of connective tissue.^[^ ^24^ ^]^ Consistent with the clinical research, the histological analysis of nilotinib‐treated mouse kidneys by hematoxylin and eosin (H&E) staining manifested the existence of organic injury (Figure 1D). The magnified images revealed that the death of glomerular vascular endothelial cells and glomerular atrophy occurred after nilotinib treatment (red arrow). In addition, we also observed that nilotinib caused renal tubular degeneration and expansion (black arrow), which corresponded to the upregulation of *Ngal* and *Kim‐1*, biomarkers of renal injury (Figure 1E,F).^[^ ^25^ ^]^ In view of previous studies indicating that apoptosis is widely implicated in the nephrotoxicity of cancer chemotherapeutic drugs, we further identified the cause of cell death by TdT mediated dUTP nick end labeling (TUNEL) staining. As a result, nilotinib induced cell apoptosis at the histological lesion location, as presented by the increasing intensity of green fluorescence (Figure 1G). Then, the mouse kidney tissue lysates were analyzed by Western blotting, and the results showed that cleaved poly(ADP‐ribose) polymerase (c‐PARP), an endogenous marker of apoptosis, was significantly upregulated after stimulation with nilotinib (Figure 1H).

**Figure 2:**
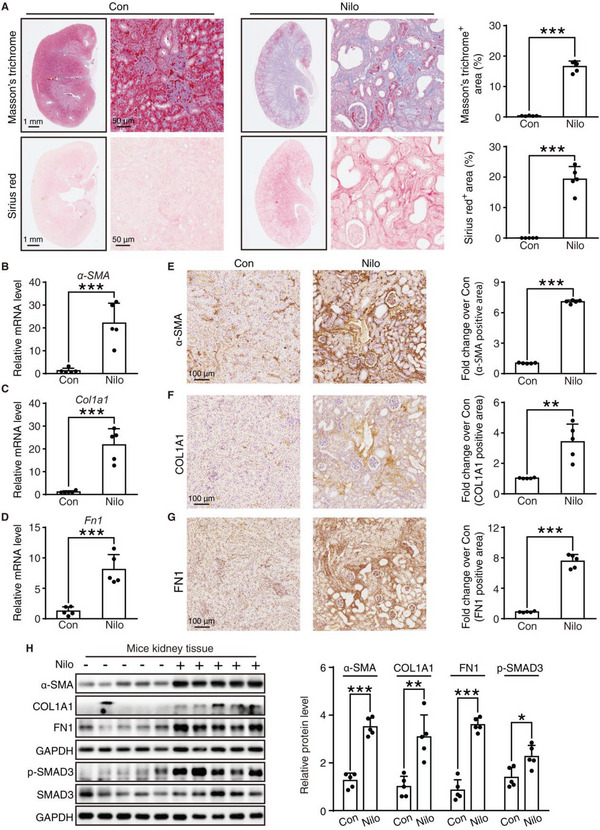
Nilotinib induces kidney fibrosis in vivo. A–H) C57BL/6J mice were administered 0.5% CMC‐Na or nilotinib (300 mg kg^−1^) by gavage for 30 days (*n* = 5 per group). A) Representative panoramic images and local zoomed images of kidney tissues with Masson's trichrome and Sirius red staining, scale bar = 1 mm or 50 µm, respectively. Quantitative analysis was performed. B–D) The mRNA expression of *α‐SMA*, *Col1a1*, and *Fn1* were analyzed by qRT‐PCR. E–G) Representative images of immunochemistry against α‐SMA, COL1A1, or FN1, scale bar = 100 µm. Relative quantitative analysis of IHC positive area was performed. H) Relative expression of α‐SMA, COL1A1, FN1, p‐SMAD3, and SMAD3 in kidney tissues were analyzed by Western blot with GAPDH as a loading control. The results are presented as the mean ± SD. The *P* value was calculated by Student's *t*‐test (unpaired, two‐tailed, two groups). *P < 0.05; **P < 0.01; ***P < 0.001.

Long‐term exposure to nephrotoxic drugs tends to cause the progression of renal fibrosis, which is characterized by the deposition of massive collagen in the glomeruli or in the tubular interstitial space.^[^ ^26^ ^]^ Then, Masson's trichrome and Sirius red staining were applied to investigate the distribution of collagen fibrils in the kidney, and the results showed that the positive area was elevated in the nilotinib‐treated group (**Figure** **2**A). There was also a significant increase in the mRNA levels of the renal fibrotic markers α‐smooth muscle actin (*α‐SMA*), collagen type I alpha 1 chain (*Col1a1*) and fibronectin 1 (*Fn1*) (Figure 2B–D). Moreover, kidney immunochemistry (IHC) analysis of α‐SMA, COL1A1 and FN1 revealed that the positive area was markedly increased after nilotinib treatment (Figure 2E–G). The levels of total protein extract of kidney tissue also confirmed renal fibrosis activation with the increment of these fibrotic markers and phosphorylated SMAD family member 3 (p‐SMAD3) (Figure 2H). Taken together, these results demonstrated severe renal fibrosis in the occurrence of nilotinib, indicating cell death, structural damage, and matrix remodeling, which is in accordance with the clinical outcome.

### 2.2 Nilotinib Induces Intrinsic Apoptosis by Promoting the Degradation of Bcl‐XL

Inspired by the in vivo results, we next proceeded to unveil the mechanism of nilotinib‐induced nephrotoxicity. Considering the histological results that glomerular vascular endothelial cells and tubular epithelial cells were the major damaged cells, we introduced human umbilical vein endothelial cells (HUVECs) and human proximal tubule epithelial cells (HK‐2) as the in vitro model of kidney injury.^[^ ^27^ ^]^ The clinically relevant Cmax of nilotinib was ≈4.27 µm according to a study on small‐molecule kinase inhibitors.^[^ ^28^ ^]^ Based on the Cmax, HUVECs and HK‐2 cells were treated with different concentrations of nilotinib (0, 4, 8, 12 µm) for 24 h. The increasing dose of nilotinib apparently changed the morphology of the cells and reduced the survival rate in both cell lines (Figure S1A,B, Supporting Information). Moreover, nilotinib greatly enhanced apoptotic rates (Figure S2A, Supporting Information) and promoted the expression of c‐PARP in a concentration‐ and time‐dependent manner (Figure S2B,C, Supporting Information). In aggregate, the above results were sufficient to verify that nilotinib induced nephrotoxicity by activating kidney cell apoptosis.

Apoptosis can be initiated by extrinsic or intrinsic signaling pathways, the latter of which has been reported to participate in multiple drug‐induced apoptosis phenotypes, such as mitochondrial dysfunction, excessive accumulation of reactive oxygen species (ROS) and DNA damage.^[^ ^29^ ^]^ First of all, JC‐1 staining combined with flow cytometry was used to detect the change in mitochondrial membrane potential (MMP), which was visualized as the ratio of green to red fluorescence signals.^[^ ^30^ ^]^ As shown in Figure S3A, Supporting Information, nilotinib decreased the MMP in a concentration‐dependent manner, which indicated enhanced mitochondrial outer membrane permeabilization and mitochondrial dysfunction. Evidence suggests that mitochondrial dysfunction usually facilitates the overproduction of ROS and that superfluous ROS cause DNA damage, which exacerbates cell death. Subsequently, DCFH‐DA staining was utilized to determine whether nilotinib induced the abnormal accumulation of ROS. The results demonstrated that nilotinib remarkably strengthened the intensity of green fluorescence, confirming the occurrence of excessive ROS (Figure S3B, Supporting Information). Furthermore, we conducted the COMET assay to assess the degree of DNA double‐strand break and discovered that nilotinib apparently induced DNA damage (Figure S4A, Supporting Information), which was also corroborated by the increased level of the DNA damage biomarker γ‐H2AX (Figure S4B,C, Supporting Information). Altogether, these results supported that nilotinib induced intrinsic apoptosis in kidney cell lines.

**Figure 3:**
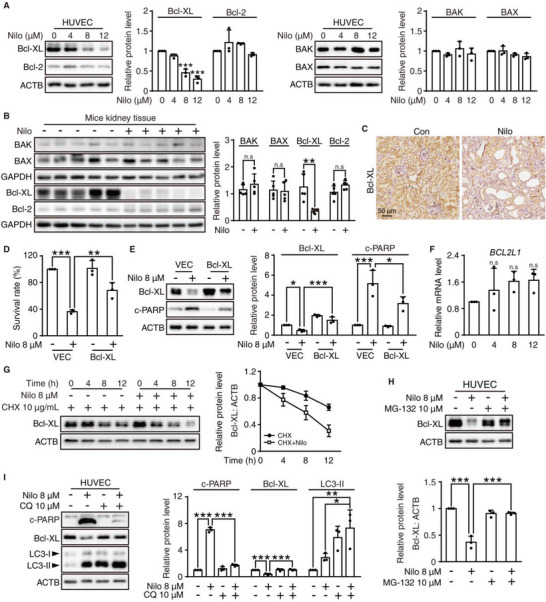
Nilotinib induces intrinsic apoptosis via promoting the degradation of Bcl‐XL. A) HUVECs were treated with nilotinib in a concentration‐dependent fashion. Relative expression of Bcl‐XL, Bcl‐2, BAK, and BAX were analyzed by Western blot. B) Relative expression of Bcl‐XL, Bcl‐2, BAK, and BAX in kidney tissues (*n* = 5 for each group) was analyzed by Western blot. C) Representative images of immunochemistry against Bcl‐XL from control group and nilotinib‐treated group, scale bar = 50 µm. D,E) HUVECs were transfected with pcDNA3.0‐Bcl‐XL plasmid or vector and exposed to 8 µm nilotinib for 24 h. *n* = 3 independent experiments. D) Cell survival rate was measured by SRB assay. E) Western blot analysis was applied to detect the relative expression of Bcl‐XL and c‐PARP. F) HUVECs were treated with nilotinib as the indicated concentrations, and the mRNA expression of *BCL2L1* was analyzed by qRT‐PCR. *n* = 3 independent experiments. G) HUVECs were treated with 10 µg mL^−1^ CHX with or without nilotinib for indicated time. Relative expression of Bcl‐XL was detected by Western blot. *n* = 3 independent experiments. H) HUVECs were treated with nilotinib for 24 h with or without 10 µm MG‐132 for 8 h before the final time point. Western blot analysis was used to detect the relative expression level of Bcl‐XL. *n* = 3 independent experiments. I) HUVECs were treated with nilotinib with or without 10 µm CQ for 24 h. Relative expression of c‐PARP, Bcl‐XL, and LC3 was analyzed by Western blot. *n* = 3 independent experiments. GAPDH or ACTB was used as a loading control. The results are presented as the mean ± SD. The *P* value was calculated by Student's *t*‐test (unpaired, two‐tailed, two groups) or one‐way ANOVA (Dunnett's multiple comparisons test). n.s = no significance; *P < 0.05; **P < 0.01; ***P < 0.001.

Because of the importance of Bcl‐2 family members in regulating cell intrinsic apoptosis, we investigated the protein levels of the major proapoptotic proteins BAK and BAX, as well as the antiapoptotic proteins Bcl‐2 and Bcl‐XL. Western blot analysis confirmed that there was no marked change in the expression of BAK, BAX, and Bcl‐2 after nilotinib stimulation, while the expression of Bcl‐XL was significantly downregulated (**Figure** **3**A and Figure S5A, Supporting Information). Similar results were observed in vivo (Figure 3B). In addition, IHC also validated the decrease in Bcl‐XL after nilotinib treatment (Figure 3C). Overexpression of Bcl‐XL in HUVECs and HK‐2 cells prominently ameliorated the nilotinib‐induced cell survival rate reduction (Figure 3D and Figure S5B, Supporting Information) and c‐PARP elevation (Figure 3E and Figure S5C, Supporting Information), suggesting that the antiapoptotic protein Bcl‐XL plays an important role in nilotinib‐induced nephrotoxicity. In order to further illustrate the relationship between Bcl‐XL reduction and the progress of nilotinib‐induced nephrotoxicity, we conducted a short‐period, including 1‐ and 2‐week nilotinib treatment in animal models. Compared to the control group, 2‐week nilotinib treatment started to show abnormalities in BUN and Scr (Figure S6A,B, Supporting Information) which were not noticeable in 1‐week treatment. The biomarkers *Ngal* and *Kim‐1* were more sensitive than BUN and Src, and the changes were observed even after 1‐week treatment (Figure S6C,D, Supporting Information). Histological analysis showed that a few apoptotic cells could be observed with cell shrinkage and the formation of eosinophilous bodies in 1 week and renal injury aggravated within 2 weeks (Figure S6E, Supporting Information). Masson's trichrome and Sirius red staining were applied to investigate the distribution of collagen fibrils in the kidney, and the results showed that mild renal fibrosis occurs in 2 weeks while no significant structure disorder in 1 week (Figure S6E–G, Supporting Information). There was also a significant increase in the protein level of the renal fibrotic marker α‐SMA after 2‐week nilotinib treatment (Figure S6H, Supporting Information). Moreover, the expression level of Bcl‐XL in kidney tissues was decreased in a time dependent manner (Figure S6I, Supporting Information) and was in according with the progression and severity of renal injury, suggesting the regulation of Bcl‐XL is the initial event in promoting nilotinib‐induced nephrotoxicity.

Thus, determining how Bcl‐XL was downregulated would contribute to presenting a strategy against nilotinib's nephrotoxicity. First, we found that nilotinib had little influence on the transcriptional level of Bcl‐XL by quantitative real‐time PCR (qRT‐PCR) assay (Figure 3F), suggesting that transcriptional inhibition was not involved in the nilotinib‐induced Bcl‐XL reduction. Then, HUVECs were pretreated with the protein biosynthesis inhibitor cycloheximide (CHX) to detect the half‐life of Bcl‐XL. The outcome indicated that nilotinib substantially reduced the half‐life of Bcl‐XL, which excluded the effect of nilotinib on the protein translation of Bcl‐XL (Figure 3G). Based on this, we speculated that the reduction in Bcl‐XL might be attributed to excessive degradation. Intracellular proteins are mostly degraded by the ubiquitin‐proteasome pathway and autophagy‐lysosome pathway. Previous studies have shown that the ubiquitin‒proteasome system is the major pathway of Bcl‐XL degradation.^[^ ^31^ ^]^ MG‐132, the 26S proteasome inhibitor, was utilized to inhibit proteasomal degradation and could apparently recover the protein level of Bcl‐XL in the context of nilotinib treatment, which suggested that nilotinib promoted the ubiquitin‒proteasome degradation of Bcl‐XL (Figure 3H). Meanwhile, the lysosomal inhibitor CQ could also rescue the protein level of Bcl‐XL to normal (Figure 3I), and it is noteworthy that CQ remarkably interfered with nilotinib‐induced cell survival and cell apoptosis while the FDA approved proteasome inhibitor bortezomib only had a slightly protective effect, even though they could both obviously regain the protein level of Bcl‐XL with nilotinib treatment (Figure S7A–C, Supporting Information). Considering the possibility and clinical feasibility of CQ as an intervention strategy for nilotinib's nephrotoxicity, it is meaningful to further investigate the underlying mechanism of CQ and identify potential intervention targets.

### 2.3 CQ restores Nilotinib‐Induced Bcl‐XL Reduction through the Regulation of USP13

CQ is a classic autophagy inhibitor that inhibits autophagic flux by reducing autophagosome–lysosome fusion.^[^ ^32^ ^]^ Since lysosomes are affected by CQ, we first introduced other classic lysosomal inhibitors, including bafilomycin A_1_ and NH_4_Cl, which can alkalize lysosomes and inhibit the degradation of substrates. However, these two lysosomal inhibitors failed to recover the protein level of Bcl‐XL when cotreated with nilotinib (Figure S8A,B, Supporting Information). We then used another autophagy inhibitor, 3‐methyladenine (3‐MA), which arrests the formation of autophagosomes to inhibit autophagy. The results revealed that 3‐MA was unable to intervene in the reduction of Bcl‐XL (Figure S8C, Supporting Information). We also found that the autophagy activator rapamycin had little effect on the protein level of Bcl‐XL (Figure S8D, Supporting Information). To eliminate the off‐target effect of tool drugs, we directly inhibited autophagy by applying siRNA targeting autophagy‐related 5 (si*ATG5*) or autophagy‐related 7 (si*ATG7*), both of which are essential for ATG conjugation and autophagosome formation. Similar results were observed and suggested that the inhibition of autophagy could not reverse the downregulation of Bcl‐XL under nilotinib treatment (Figure S8E,F, Supporting Information). Thus, we concluded that autophagy was not involved in the nephrotoxicity of nilotinib and that the protective function of CQ did not rely on autophagy inhibition. Previous studies also demonstrated that CQ exerted autophagy‐independent effects via the regulation of NOTCH1 and SIGMAR1.^[^ ^22^ ^]^ Hence, we investigated whether NOTCH1 or SIGMAR1 is involved in the protective effect of CQ by siRNA. Western blot analysis showed that CQ could still attenuate nilotinib‐induced cell apoptosis after the silencing of *NOTCH1* or *SIGMAR1*, indicating that the protective function of CQ did not rely on known targets (Figure S9A,B, Supporting Information).

**Figure 4:**
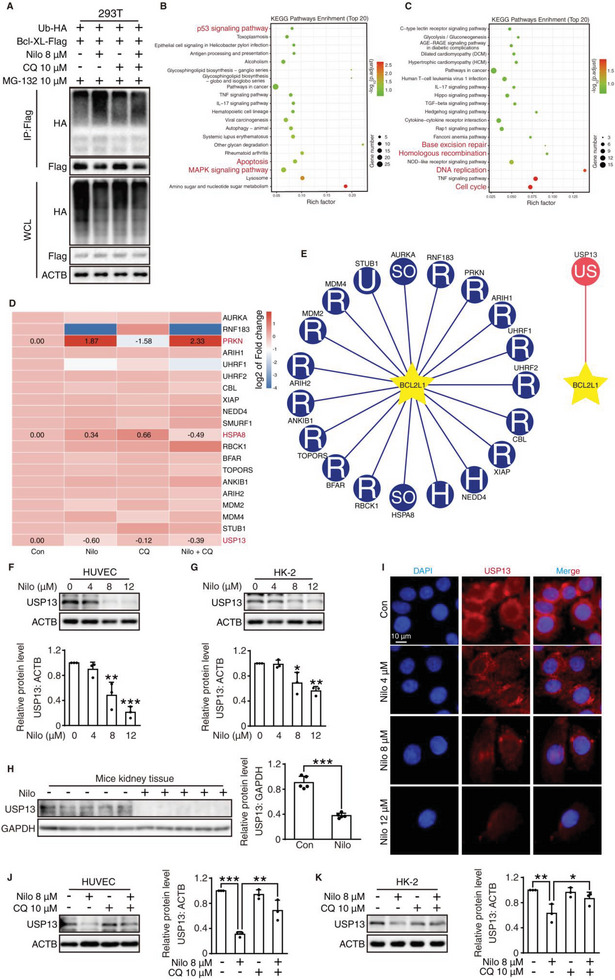
CQ restores nilotinib‐induced Bcl‐XL reduction through the regulation of USP13. A) HEK293T cells were transfected with Bcl‐XL‐FLAG and Ubiquitin‐HA plasmid for 24 h, then HEK293T cells were treated as indicated. Bcl‐XL was immunoprecipitated by using anti‐FLAG beads and ubiquitylated Bcl‐XL was detected using an anti‐HA antibody. B‐D) HUVECs samples from control, nilotinib‐, CQ‐, and nilotinib plus CQ‐treated groups were subjected to RNA‐seq analysis. KEGG enrichment analysis of B) upregulated genes and C) downregulated genes between control and nilotinib‐treated group. D) Heatmap showing fold changes of E3 ligases and deubiquitinases among RNA‐seq analysis. E) Network for predicted E3 ligases and deubiquitinases of Bcl‐XL in UbiBrowser web services. The selected genes according to UbiBrowser results were marked in red. F,G) HUVECs and HK‐2 cells were treated with 0, 4, 8,12 µm nilotinib for 24 h. Western blot analysis was applied to detect the relative expression of USP13. *n* = 3 independent experiments. H) Relative expression of USP13 in kidney tissues (*n* = 5 for each group) was detected and analyzed by Western blot. I) HK‐2 cells were treated with 0, 4, 8, 12 µm nilotinib for 24 h. Representative images of HUVECs stained with USP13 (red) and DAPI (blue) were shown by immunofluorescence assay, scale bar = 10 µm. J,K) HUVECs and HK‐2 cells were treated with nilotinib with or without 10 µm CQ for 24 h. Relative expression of USP13 was analyzed by Western blot. *n* = 3 independent experiments. For Western blot, ACTB or GAPDH was used as a loading control. The results are presented as the mean ± SD. *P* value was calculated by one‐way ANOVA (Dunnett's multiple comparisons test). *P < 0.05; **P < 0.01; ***P < 0.001.

The above results have proven that nilotinib promoted the overdegradation of Bcl‐XL independently of autophagy; therefore, we hypothesized that CQ might affect the ubiquitination modification of Bcl‐XL and further regulate the protein level of Bcl‐XL. By coexpressing exogenous ubiquitin and Bcl‐XL in human embryonic kidney 293 T (HEK293T) cells, we confirmed that nilotinib increased the level of ubiquitylated Bcl‐XL, which could be reversed by CQ (**Figure** **4**A). Considering nilotinib as a tyrosine kinase inhibitor, we then proceeded to investigate the changes in protein and signaling pathways to identify potential Bcl‐XL stability regulators by performing RNA sequencing (RNA‐seq). Kyoto Encyclopedia of Genes and Genomes (KEGG) signaling pathway analysis between the nilotinib treatment group and the control group showed that genes involved in the apoptosis and DNA repair pathways were enriched in the upregulated and downregulated groups, respectively, which is consistent with our findings (Figure 4B,C). Given that E3 ligases and DUBs are the major components of the ubiquitination regulation system, we then used UbiBrowser, an integrated bioinformatics platform, to predict the interactions between substrates and E3 ligases/DUBs.^[^ ^33^ ^]^. Based on the predictive outcomes, we searched for alterable candidates in the results of RNA‐seq according to the following standards: 1) the level of E3 ligase was upregulated in the nilotinib treatment group and reduced when combined with CQ. 2) DUBs that were downregulated in the presence of nilotinib and increased when combined with CQ. As a result, three genes satisfied the standards: PRKN, HSPA8, and USP13 (Figure 4D,E). Subsequent Western blot analysis revealed that only USP13, whose protein level was decreased under nilotinib treatment both in vitro and in vivo (Figure 4F–H and Figure S10A,B, Supporting Information), met our criteria. Notably, the known regulated proteins of USP13, such as p53 and MCL‐1,^[^ ^17^, ^18^ ^]^ were also reduced in the presence of nilotinib (Figure S11A,B, Supporting Information). Immunofluorescence assays further identified that nilotinib markedly downregulated the expression of USP13 (Figure 4I). When combined with CQ, the results showed that CQ significantly reversed the nilotinib‐induced downregulation of USP13 (Figure 4J,K); thus, USP13 may be a novel regulated protein of CQ.

### 2.4 USP13 Interacts with and Stabilizes Bcl‐XL

**Figure 5:**
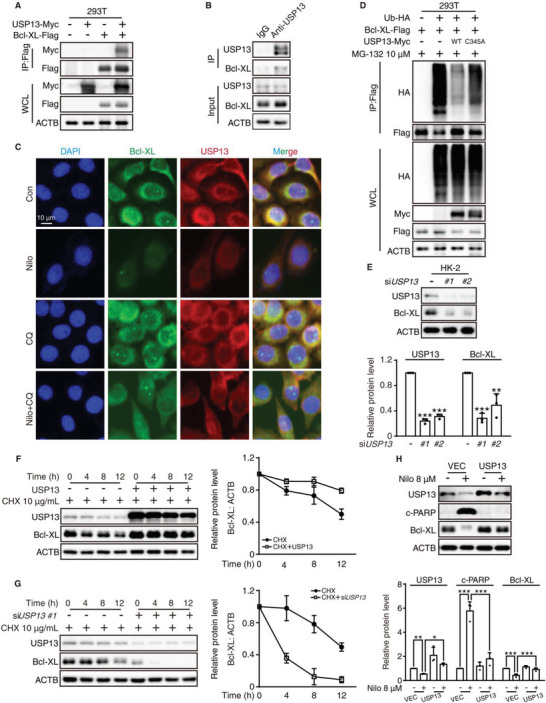
USP13 interacts with and stabilizes Bcl‐XL. A) HEK293T cells were transfected with or without USP13‐Myc, Bcl‐XL‐Flag. Cells were treated with 10 µm MG‐132 for 12 h. Bcl‐XL was immunoprecipitated with anti‐Flag beads. USP13 was detected using an anti‐Myc antibody. B) Endogenous USP13 from HK‐2 cells was immunoprecipitated with anti‐USP13 antibody and Bcl‐XL was analyzed by Western blot. C) HK‐2 cells were treated with nilotinib with or without 10 µm CQ for 24 h. Representative images of HK‐2 cells stained with USP13 (red), Bcl‐XL (green) and DAPI (blue) were shown by immunofluorescence assay, scale bar = 10 µm. D) Bcl‐XL‐Flag was co‐transfected with wild‐type USP13‐Myc or mutant USP13‐C345A‐Myc, with or without Ub‐HA into HEK293T. Cells were treated with 10 µm MG‐132 for 8 h and Bcl‐XL was immunoprecipitated by using anti‐Flag beads. Ubiquitylated Bcl‐XL was detected using an anti‐HA antibody. E,F) Western blot analysis of lysates from HK‐2 cells transfected with control siRNA (negative control) or siRNA targeting USP13. *n* = 3 independent experiments. E) Relative expression of USP13 and Bcl‐XL was analyzed. F) Cells were then treated with 10 µg mL^−1^ CHX for different durations and collected for Western blot to detect the relative expression of USP13 and Bcl‐XL. G) HK‐2 cells were transfected with vector or USP13 and treated with 10 µg mL^−1^ CHX for different durations. Relative protein level was detected by Western blot. *n* = 3 independent experiments. H) HK‐2 cells were transfected with USP13 plasmid or vector and exposed to 8 µm nilotinib for 24 h. Relative expression of USP13, c‐PARP, and Bcl‐XL were analyzed by Western blot. *n* = 3 independent experiments. For Western blot, ACTB was used as a loading control. The results are presented as the mean ± SD. The *P* value was calculated by one‐way ANOVA (Dunnett's multiple comparisons test). *P < 0.05; **P < 0.01; ***P < 0.001.

The correlation between USP13 and Bcl‐XL has not yet been reported; thus, we first applied coimmunoprecipitation analysis to investigate the direct interaction between USP13 and Bcl‐XL. By coexpressing USP13‐Myc and Bcl‐XL‐Flag in HEK293T cells, we discovered that USP13 could be detected in Bcl‐XL immunoprecipitates after pull down by anti‐Flag beads (**Figure** **5**A). Meanwhile, endogenous Bcl‐XL could combine with USP13 in HK‐2 cells (Figure 5B). We then performed an immunofluorescence assay to validate the colocalization of USP13 and Bcl‐XL. The colocalization was greatly diminished by nilotinib treatment but recovered after combination treatment with CQ (Figure 5C). Because USP13 is a canonical DUB, we constructed the catalytically inactive mutant USP13‐C345A plasmid via site mutation. Further analysis revealed that wild‐type USP13 but not mutant USP13 markedly reduced the amount of ubiquitinated Bcl‐XL (Figure 5D). Subsequently, we proceeded to examine whether USP13 dictated the stability of Bcl‐XL under the basal state or after treatment with nilotinib. First, we silenced *USP13* with siRNA in HK‐2 cells, and Western blot analysis revealed that *USP13* knockdown led to the downregulation of Bcl‐XL (Figure 5E). Furthermore, overexpression or knockdown of *USP13* resulted in an increase or decrease in the half‐life of Bcl‐XL, respectively (Figure 5F,G). Then, we investigated the effect of USP13 on Bcl‐XL in the presence of nilotinib, and the results revealed that overexpression of USP13 completely reversed nilotinib‐induced downregulation of Bcl‐XL and significantly reversed cell death caused by nilotinib (Figure S12, Supporting Information and Figure 5H). Conclusively, the above findings confirmed that USP13 interacted with and stabilized Bcl‐XL under both physiological and pathological conditions, thus involving in nilotinib‐induced nephrotoxicity.

### 2.5 CQ Relieves Nilotinib‐Induced Nephrotoxicity In Vitro

The studies presented above indicated that CQ is a potential intervention strategy for nilotinib‐induced nephrotoxicity. To prove the feasibility of CQ for clinical use, we investigated the effect of CQ on the nilotinib‐induced apoptosis‐related phenotype. We found that CQ reduced the elevation of the nilotinib‐induced cell apoptotic rate (Figure S13A, Supporting Information), remarkably reversed the nilotinib‐induced decrease in MMP (Figure S13B, Supporting Information) and inhibited nilotinib‐induced intracellular ROS accumulation (Figure S13C, Supporting Information). Furthermore, we assessed DNA damage by COMET assay and the protein level of γ‐H2AX by Western blot, confirming that CQ was able to reverse nilotinib‐induced DNA damage (Figure S14A,B, Supporting Information). Notably, CQ did not affect the anticancer effect of nilotinib in the BCR‐ABL1^+^ cell Line K562 (Figure S15A, Supporting Information). These results jointly demonstrated that CQ could rescue nilotinib‐induced cell apoptosis and was expected to become an attractive intervention strategy for nilotinib's nephrotoxicity in clinics.

### 2.6 HCQ Rescues Nilotinib‐Induced Nephrotoxicity

**Figure 6:**
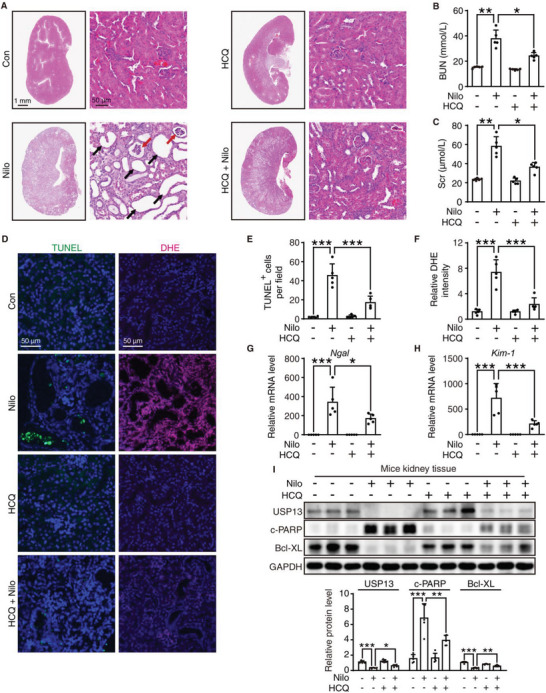
HCQ relieves nilotinib‐induced kidney cells apoptosis and renal injury in vivo. A–I) C57BL/6J mice were administered 0.5% CMC‐Na, nilotinib (300 mg kg^−1^), HCQ (30 mg kg^−1^) or nilotinib plus HCQ by gavage for 30 days (*n* = 5 per group). Kidneys and serum were harvested. A) Representative panoramic images and local zoomed images of kidney tissues with hematoxylin and eosin staining (H&E), Scale bar = 1 mm or 50 µm, respectively. Red arrows indicated the death of glomerular vascular endothelial cells and glomerular atrophy. Black arrows indicated the degeneration and expansion of renal tubular. B) BUN and C) Scr levels were analyzed. D) Fluorescence microscope images of kidney tissues stained with TUNEL or DHE. Scale bar = 50 µm. Quantitative analysis was performed to detect E) apoptotic cells and F) DHE intensity. G,H) The mRNA expression of *Ngal* and *Kim‐1* were analyzed by qRT‐PCR. I) Relative expression of c‐PARP, USP13, and Bcl‐XL were analyzed by Western blot. GAPDH was used as a loading control. The results are presented as the mean ± SD. The *P* value was calculated by one‐way ANOVA (Dunnett's multiple comparisons test). *P < 0.05; **P < 0.01; ***P < 0.001.

Inspired by the in vitro results, we intended to evaluate whether CQ ameliorated the nephrotoxicity of nilotinib in vivo. Considering the potential risk of CQ inducing retinopathy and cardiotoxicity,^[^ ^34^ ^]^ we proposed to substitute its derivative HCQ, whose adverse events were reported less frequently than CQ. We then detected the effect of HCQ on nilotinib‐induced nephrotoxicity in vitro. HCQ possessed similar effects as CQ, including an influence on the anticancer response (Figure S15B, Supporting Information) and the related protein levels (Figure S16A,B, Supporting Information). The outcome of H&E staining demonstrated that the nilotinib‐plus‐HCQ group experienced less glomerular atrophy and tubular dilatation than the nilotinib group (**Figure** **6**A). Meanwhile, the blood biochemical examination results showed that HCQ treatment significantly reversed the nilotinib‐induced elevation of BUN and Scr (Figure 6B,C). In addition, TUNEL and dihydroethidium (DHE) staining indicated that kidney cell apoptosis and oxidative stress induced by nilotinib were remarkably ameliorated when combined with HCQ (Figure 6D–F). We also applied qRT‐PCR assays to detect the gene expression of *Ngal* and *Kim‐1*, and the results showed that combination treatment with HCQ resulted in less gene expression of *Ngal* and *Kim‐1* than treatment with nilotinib alone, which indicated effective improvement of renal injury (Figure 6G,H). Moreover, in line with the in vitro results, Western blot analysis revealed that HCQ rescued nilotinib‐induced nephrotoxicity by upregulating the expression of USP13 and restoring the protein level of the antiapoptotic protein Bcl‐XL, ultimately reversing nilotinib‐induced kidney cell apoptosis (Figure 6I).

**Figure 7:**
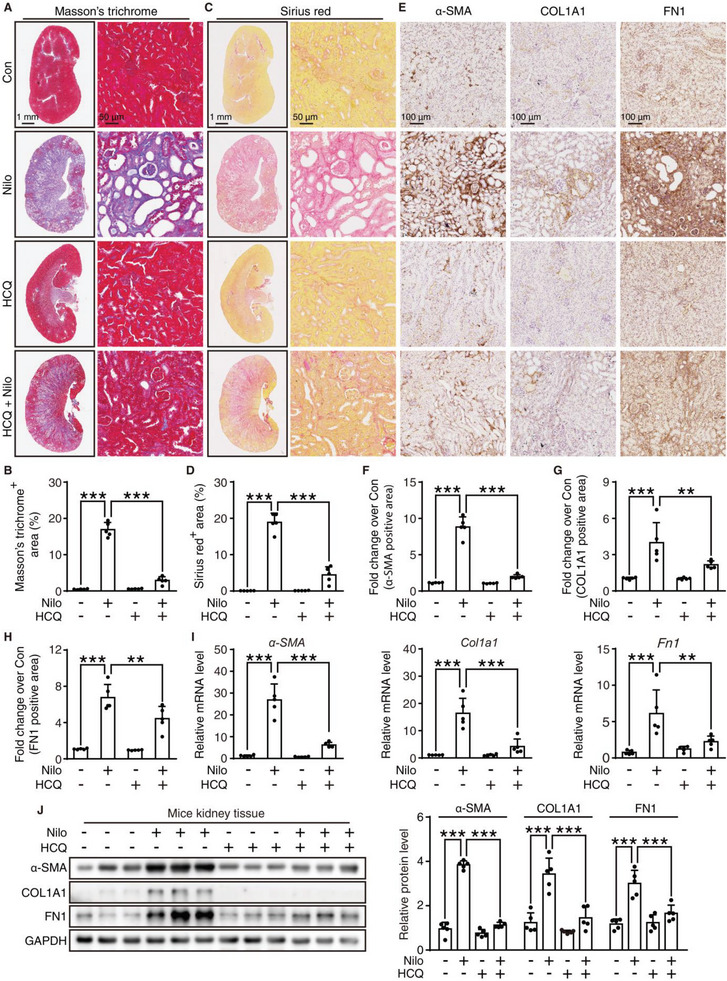
HCQ rescues nilotinib‐induced kidney fibrosis in vivo. A–J) C57BL/6J mice were administered 0.5% CMC‐Na, nilotinib (300 mg kg^−1^), HCQ (30 mg kg^−1^) or nilotinib plus HCQ by gavage for 30 days (*n* = 5 per group). Kidneys and serum were harvested. A–D) Representative panoramic images and local zoomed images of kidney tissues with Masson's trichrome (A) and Sirius red staining (C), scale bar = 1 mm or 50 µm, respectively. Quantitative analysis was performed to detect Masson's trichrome positive area (B) and Sirius red staining positive area (D). E) Representative images of immunochemistry against α‐SMA, COL1A1, or FN1, scale bar = 100 µm. Relative quantitative analysis of F) α‐SMA positive area, G) COL1A1 positive area, and H) FN1 positive area were performed. I) The mRNA expression of *α‐SMA*, *Col1A1*, and *Fn1* were analyzed by qRT‐PCR. J) Relative expression of α‐SMA, COL1A1, and FN1 were analyzed by Western blot. GAPDH was used as a loading control. The results are presented as the mean ± SD. The *P* value was calculated by one‐way ANOVA (Dunnett's multiple comparisons test). n.s = no significance; *P < 0.05; **P < 0.01; ***P < 0.001.

Then, we investigated whether combination treatment with HCQ can attenuate renal fibrosis. Compared to the nilotinib treatment group, HCQ administration significantly lessened the deposition of collagen in the glomeruli or tubular interstitial space, which was confirmed by Masson's trichrome (**Figure** **7**A,B) and Sirius red staining (Figure 7C,D). We then performed an IHC assay to detect the amount of fibrotic markers, including α‐SMA, COL1A1, and FN1, and the results revealed that HCQ was able to decrease the upregulation of α‐SMA, COL1A1, and FN1 protein levels in the nilotinib treatment group, especially in the tubulointerstitial area (Figure 7E–H). HCQ was also shown to reverse the nilotinib‐induced elevated mRNA levels of *α‐SMA*, *Col1a1*, and *Fn1* (Figure 7I), which was in accordance with the Western blot analysis (Figure 7J). Moreover, the upregulated p‐SMAD3 resulting from nilotinib was rescued to basal levels when combined with HCQ (Figure S17, Supporting Information). Consequently, these results were sufficient to support that CQ analogs could be an attractive intervention strategy for nilotinib‐induced nephrotoxicity in clinics.

## 3 Discussion

**Figure 8:**
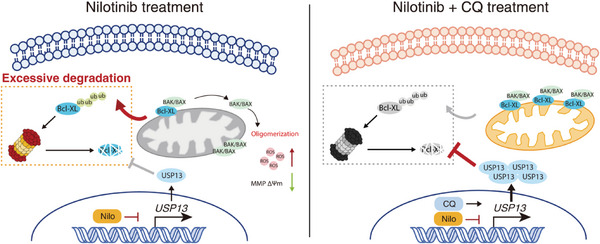
Schematic diagrams of the mechanism of nilotinib's nephrotoxicity and CQ's renoprotective effects. Nilotinib down‐regulates the expression of USP13 via transcriptional repression and hence prevents the influence of USP13 on inhibition of Bcl‐XL's ubiquitination degradation. Excessive degradation of Bcl‐XL breaks the balance between Bcl‐2 family members, induces the loss of mitochondrial membrane potential and ROS accumulation. By activation the transcription of USP13, CQ exerts a significant anti‐apoptotic function against nilotinib and ameliorates nilotinib's nephrotoxicity.

In this study, we first uncovered that kidney cell apoptosis mediated by the intrinsic signaling pathway plays a pivotal role in the nephrotoxicity of nilotinib. We revealed that excessive ubiquitin–proteasome pathway degradation of the antiapoptotic protein Bcl‐XL causes kidney cell apoptosis due to a decrease in the deubiquitinase USP13. More importantly, we found that CQ, a classic autophagy inhibitor, can interfere with the nephrotoxicity of nilotinib in an autophagy‐independent manner and that USP13 may be the functional target of CQ in protecting against nilotinib‐induced kidney cell apoptosis (**Figure** **8**).

Bcl‐2 family members function as key regulators of intrinsic apoptosis and are critical for the maintenance of major organ systems.^[^ ^35^ ^]^ A previous study revealed that drug‐induced nephrotoxicity is closely associated with alterations in Bcl‐2 family members.^[^ ^36^ ^]^ Herein, we discovered that the nephrotoxicity of nilotinib is highly related to the marked downregulation of the antiapoptotic protein Bcl‐XL by accelerating its ubiquitin‒proteasome degradation; however, we found that nilotinib has no obvious effect on other major members of the Bcl‐2 protein family, such as Bcl‐2, BAX, and BAK, indicating a special mechanism for the nephrotoxicity of nilotinib compared with other apoptosis‐related drugs. Compared with other antiapoptotic proteins, Bcl‐XL exerts a more significant antiapoptotic effect because of its involvement in the retrograde transport of BAX, which enables mitochondrial‐anchored BAX to return to the cytoplasm.^[^ ^37^ ^]^ In addition, Bcl‐XL was also reported to sustain cellular Ca^2+^ homeostasis and inhibit Ca^2+^‐induced cell apoptosis.^[^ ^38^ ^]^ To date, an increasing number of studies have demonstrated the relevance between Bcl‐XL and drug‐induced nephrotoxicity, and the downregulation of Bcl‐XL occurs in cisplatin‐, cyclosporin A‐, and Adriamycin‐induced renal injury.^[^ ^39^ ^]^ In addition, the intracellular level of Bcl‐XL is also associated with other kidney diseases, such as proteinuric kidney disease, IgA nephropathy and ischemia‒reperfusion renal injury.^[^ ^40^ ^]^ Regarding the reason for the downregulation of Bcl‐XL, the current study has mainly attributed it to the harmful factors’ effect on the transcriptional level or protein stability of Bcl‐XL. The ubiquitin‒proteasome pathway is widely described to be involved in the regulation of Bcl‐XL protein stability. The major E3 ligases, for example, RNF183 and PRKN, were reported to promote its ubiquitination and degradation.^[^ ^13^, ^31^ ^]^ However, there is no relevant report on the involvement of DUBs in the regulation of Bcl‐XL stability. We have demonstrated that USP13 could deubiquitinate and stabilize Bcl‐XL and play a pivotal role in nilotinib's nephrotoxicity. Hence, our work will not only clarify the molecular mechanism of nilotinib's nephrotoxicity but also enrich the regulatory system of Bcl‐XL protein stability.

USP13 has been previously implicated in the development of multiple cancers. For instance, USP13 was shown to be upregulated in lung cancer and ovarian cancer, and its intracellular level was associated with the progression of malignant tumors.^[^ ^18^, ^41^ ^]^ In addition, USP13 was also found to be involved in renal cell carcinoma,^[^ ^19^ ^]^ which indicated the potential relevance between USP13 and kidney disease. However, the role of USP13 in other kidney diseases is still obscure. Considering that another antiapoptotic Bcl‐2 family member, MCL‐1, is also the known substrate of USP13 and whose expression was reduced after nilotinib treatment, it is imperative to further investigate whether MCL‐1 participates in the nephrotoxicity of nilotinib because overexpression of Bcl‐XL only partially reversed nilotinib‐induced kidney cell apoptosis.

CQ or its derivative HCQ is the classic drug in clinics for the treatment of malaria and autoimmune diseases.^[^ ^20^ ^]^ Because CQ is a well‐recognized lysosome inhibitor and is considered an important tool drug in the field of autophagy, researchers usually attribute CQ's pharmacological effect to autophagy inhibition but rarely focus on the autophagy‐independent role of CQ. In this study, we confirmed that autophagy inhibition was not involved in CQ's intervention effect against nilotinib's nephrotoxicity, and RNA‐seq analysis and UbiBrowser were applied to identify USP13 as a new regulated target of CQ. As an immunomodulator, CQ is also recommended for the treatment of lupus nephritis, and a mechanistic study revealed that CQ acts on the innate immune system by blocking toll like receptor signaling and reducing the production of proinflammatory cytokines.^[^ ^42^ ^]^ Hence, whether USP13 plays an important role in CQ's treatment of lupus nephritis deserves future work.

As the second generation of BCR‐ABL1 inhibitors, nilotinib is extensively used in clinics for the treatment of CML. In addition, it has been proven in multiple clinical trials to improve cognitive and athletic ability in patients with Parkinson's and Alzheimer's disease and is expected to become the first drug to treat neurodegenerative diseases.^[^ ^43^ ^]^ However, nilotinib has been labeled Black Box Warning by the FDA because of its severe cardiotoxicity, which is exacerbated by nilotinib‐induced nephrotoxicity due to disorders of electrolyte and humoral regulation.^[^ ^44^ ^]^ It is possible to effectively ameliorate the risk of cardiotoxicity and prolong the time of medical use of nilotinib by solving nephrotoxicity. Though multiphasic clinical trials failed to identify serious renal toxicity, real‐life experience with nilotinib has evidenced some uncommon renal adverse effects due to long‐term exposure and the post‐marketing pharmacovigilance system. Alongside the renal failure and fluid retention signals, the nephrotic syndrome and renal artery stenosis were identified as new safety signals for nilotinib based on VigiBase data and the monitoring of long‐term use renal safety of nilotinib is imperative.^[^ ^45^ ^]^ This study proposed an effective intervention strategy, and our work is of great clinical significance. Notably, we also investigated whether other BCR‐ABL1 inhibitors, including imatinib, dasatinib, bosutinib, and ponatinib, could cause the same change in kidney cells as nilotinib. However, the results showed that these four BCR‐ABL1 inhibitors did not cause severe nephrotoxicity and could not induce the downregulation of USP13 (Figure S18, Supporting Information), which proved that nilotinib‐induced nephrotoxicity was independent of its inhibition of pharmacological targets. Concerning the fact that some previous studies suggest that nilotinib was able to alleviate both chronic and acute kidney injury via its inhibitory effect on platelet derived growth factor receptor,^[^ ^46^ ^]^ we found that the nilotinib dosage recommended in their studies was much lower than that clinically used. Moreover, there are multiple major causes of kidney fibrosis, leading to the potential of the drug's double‐edge sword function in the progression of fibrosis. For example, sunitinib was reported to improve liver fibrosis in the cirrhotic process;^[^ ^47^ ^]^ nevertheless, its hepatotoxicity is warning by the FDA, and reports about its severe hepatic adverse events exist.^[^ ^48^ ^]^ We cannot deny the potential intervention effect of nilotinib on fibrosis; however, nilotinib may promote an excessive imbalance and dominantly acts as a nephrotoxicity accelerator when used alone.

## 4 Conclusion

In summary, the present study demonstrates for the first time that nilotinib represses the expression of USP13 and further accelerates the degradation of the antiapoptotic protein Bcl‐XL, leading to nephrotoxicity. Moreover, we provide a clinically used drug, CQ or HCQ, as the intervention strategy for nilotinib's nephrotoxicity. The mechanistic study of CQ's intervention effect reveals that CQ relieves nilotinib's nephrotoxicity independent of autophagy inhibition and that USP13 is a novel regulated target of CQ. This research also provides new insight into the molecular mechanism by which USP13 regulates the protein stability of Bcl‐XL, which relies on catalytic activity. Moreover, we established a pathological model by nilotinib treatment that mimics the phenotype of chronic kidney disease well. Based on this model, we may have additional insights into the progression of renal injury and fibrosis.

## 5 Experimental Section

### Animals

6–8 weeks male C57BL/6J mice were purchased from the Shanghai Slac Laboratory Animal Company Limited (Shanghai, China). All mice procedures were performed according to the Institutional Animal Care and Use Committee protocol of Innovation Institute for Artificial Intelligence in Medicine of Zhejiang University. All experiments were conducted with protocols approved by Innovation Institute for Artificial Intelligence in Medicine of Zhejiang University (approval No. DW202203301824 and DW202305091849). The mice were housed in the animal facilities with a 12 h light dark cycle, food and water available ad libitum. Before performing in vivo experiment, 1 week period was provided to allow the animals to adapt to the laboratory environment. Nilotinib (CAS: 641571‐10‐0, HEOWNS, Tianjin, China) and HCQ (CAS: 747‐36‐4, TCI, Shanghai, China) were dissolved in 0.5% sodium carboxymethyl cellulose (CMC‐Na) (419273, Sigma‐Aldrich, St. Louis, USA) to obtain stock. In the study of nilotinib's nephrotoxicity, the mice were treated with 0.5% CMC‐Na, 300 mg kg^−1^ nilotinib daily via intragastric administration for 7, 14, or 30 days; in the study of HCQ's intervention effects, the mice were treated with 0.5% CMC‐Na, 300 mg kg^−1^ nilotinib, 30 mg kg^−1^ HCQ, or nilotinib plus HCQ daily via intragastric administration for 30 days.

### Blood Biochemistry Analysis

The blood was collected into the 1.5 mL collection tubes by bleeding mice from the retro‐orbital plexus under isoflurane anesthesia. Then, the blood samples were stood at room temperature for more than 1 h. At the end, the whole blood samples were centrifuged at 4000 rpm for 10 min to collect the serum for the detection of Scr and BUN.

### Histopathological and Immunohistochemical Analysis

Mice were sacrificed to harvest the kidneys. One side of kidney was divided into two parts. One of them was quickly frozen in liquid nitrogen and stored at −80 °C and the other was fixed in 10% phosphate‐buffered formalin (F8775, Sigma‐Aldrich, St. Louis, USA) (pH = 7.4) and embedded in paraffin before cut into 4 µm slices. The tissue sections were processed and stained with H&E, Masson's trichrome or Sirius red by standard protocols. For immunohistochemical analysis, sections were stained with the following primary antibody: α‐SMA (ab124964, Abcam, Cambridge, UK), COL1A1 (84336S, Cell Signaling Technology, Boston, USA), FN1 (ab23750, Abcam, Cambridge, UK), Bcl‐XL (ET1603‐28, Huabio, Hangzhou, China). Finally, all stained slides were recorded and analyzed on a pathological section scanner (HS6, SUNNY INSTRUMENT CO., LTD, Ningbo, China).

### TUNEL Assay

One Step TUNEL apoptosis assay kit (C1088, Beyotime, Shanghai, China) was applied to detect cell apoptosis in kidney section according to manufacturer's instruction. Briefly, the tissue sections were pretreated with Proteinase K (ST532, Beyotime, Shanghai, China) working solution after dewaxing and rehydration. Then, TUNEL detection solution was added on the slides and incubated with tissues samples for 60 min at 37 °C in a humidified chamber in the dark. Finally, nuclei were stained with 4′,6‐diamidino‐2‐phenylindole (DAPI) (D212, Dojindo, Kumamoto, Japan) and the TUNEL signals were observed with fluorescence microscope (IX81‐FV1000, Olympus, Tokyo, Japan).

### DHE Staining

ROS production in kidney tissues was detected with DHE (S0063, Beyotime, Shanghai, China). DHE was dissolved in dimethyl sulfoxide at a stock concentration of 5 mm and diluted with phosphate buffered saline (PBS) to 1 µm before using. The kidney slides were incubated with DHE in a light‐protected humidified box at room temperature for 30 min. Nuclei were stained with DAPI and imaged with a fluorescence microscope (IX81‐FV1000, Olympus, Tokyo, Japan).

### Cell Culture and Treatment

HUVECs, HK‐2 cells, and HEK293T cells were purchased from the Institute of Biochemistry and Cell Biology (Shanghai, China). K562 leukemia cells were purchased from National Biomedical Cell Line Resource Center (Peking, China). All cell lines except HEK293T (maintained in DMEM (12800, Gibco, Carlsbad, USA)) were maintained in RPMI‐1640 (31800, Gibco, Carlsbad, USA) supplemented with 10% fetal bovine serum (SH30396.03, Hyclone, Logan, USA), 100 U mL^−1^ penicillin and 100 µg mL^−1^ streptomycin in a humidified atmosphere with 5% CO_2_ and 95% air at 37 °C.

Cycloheximide (CAS: 66‐81‐9), CQ (CAS: 54‐05‐7), rapamycin (CAS: 53123‐88‐9), and bafilomycin A_1_ (CAS: 88899‐55‐2) were purchased from Selleck Chemicals (Shanghai, China). MG‐132 (CAS: 133407‐82‐6), 3‐methyladenine (3‐MA) (CAS: 5142‐23‐4), and NH_4_Cl (CAS: 12125‐02‐9) were purchased from Sigma‐Aldrich (St. Louis, USA). HCQ (CAS: 747‐36‐4) was purchased from TCI (Shanghai, China). Bortezomib (CAS: 179324‐69‐7) was purchased from MedChemExpress (Shanghai, China). Throughout the experiments (if not stated otherwise), HUVECs and HK‐2 cells were treated with 8 µm nilotinib for the indicated time periods or with 0, 4, 8, or 12 µm nilotinib for 24 h. In selected samples, 10 µg mL^−1^ CHX, 10 µm CQ, 10 µm HCQ, 10 mm NH_4_Cl, 10 nm bafilomycin A_1_, 2 mm 3‐MA, 0.5 µm rapamycin, and 10 nm bortezomib were added and incubation for 24 h. In some samples, 10 µm MG‐132 was added 8 h before cell collection.

### Cell Survival Rate Analysis

The sulforhodamine B (SRB) colorimetric assay was used to assess the cell survival rate. Briefly, cells were plated in 96‐well plates (5 × 10^3^ cells per well for HUVECs and 1 × 10^4^ for HK‐2) or 12‐well plates (8 × 10^4^ cells per well for HUVECs and 1.5 × 10^5^ cells per well for HK‐2) and left for 24 h of free growth. After 24 h of drug exposure, the cells were fixed with 10% (w/v) trichloroacetic acid (CAS: 76‐03‐9, Aladdin Biochemical Technology Co., LTD., Shanghai, China) for over 1 h at 4 °C and stained with 0.4% SRB (CAS: 3520‐42‐1, Sigma‐Aldrich, St. Louis, USA) (w/v; dissolved in 1% acetic acid (CAS: 64‐19‐7, Sinopharm Chemical Reagent Co., LTD., Shanghai, China) aqueous solution) for 30 min. Unbound SRB was removed by washing with 1% acetic acid and bound stains were solubilized with 10 mm Tris‐base. The absorbance of all the wells were measured by Multiskan Spectrum instrument (Thermo Electron Corporation, Georgia, USA) at a single wavelength of 510 nm. The cell survival rate was calculated for each well as follows: (the absorbance of treated cells/the absorbance of control cells) × 100%.

### Cell Viability Analysis

The CCK‐8 assay was applied to detect cell viability. In brief, cells were plated in 96‐well plates (5 × 10^3^ cells per well for K562) and left for 24 h of free growth. After 24 h of drug exposure, 20 µL of CCK‐8 (C0037, Beyotime, Shanghai, China) was added to per well of 96‐well plate and incubated at 37 °C for 2 h. The Multiskan Spectrum instrument was used to measure the OD value at 450 nm. The wells with cell‐free 1640 complete medium was set as the negative control group to eliminate the influence of environmental background. The cell viability was calculated for each well as follows: (the OD value of treated cells − the OD value of negative control)/(the OD value of control cells‐the OD value of negative control) × 100%.

### Flow Cytometric Analysis

For Annexin V‐PI staining, cells were seeded in 6‐well plates (2 × 10^5^ cells per well for HUVECs and 3 × 10^5^ cells per well for HK‐2) and left for 24 h of free growth. Then, cells were treated with drugs for 24 h. At the end of the incubation period, cells were harvested and washed with PBS. The FITC Annexin V Apoptosis Detection Kit I (556547, BD Pharmingen, Franklin Lakes, USA) was applied to detect the apoptotic rate by using a CytoFLEX cytometer (B53015, Beckman Coulter, Brea, USA) according to manufacturer's instruction.

For JC‐1 staining, cells were treated and harvested as mentioned before. Then, cells were incubated with JC‐1 probes (C2006, Beyotime, Shanghai, China) for 20 min at 37 °C. At the end of the incubation period, the cells were washed with PBS twice and then resuspended in 500 µL PBS. The change in mitochondrial membrane potential was detected by a CytoFLEX cytometer.

### ROS Measurement

Intracellular ROS were analyzed by using a ROS detection kit (S0033S, Beyotime, Shanghai, China) according to manufacturer's instructions. In brief, cells were harvested and incubated with DCFH‐DA probes for 20 min at 37 °C. During the incubation, the incubation system should be mixed upside and down every 3–5 min. At the end, the cells were washed with FBS‐free 1640 culture medium to remove the unbound DCFH‐DA probes and resuspended in 500 µL 1640 culture medium. The intracellular level of ROS was detected by a CytoFLEX cytometer, or imaged by fluorescence microscope.

### Immunofluorescence Assay

After specific treatment, cultured cells grown on 96‐well plate were washed with PBS twice and incubated with 4% paraformaldehyde (P6148, Sigma‐Aldrich, St. Louis, USA) for 20 min. Then, the cells were permeabilized with the treatment of 0.1% Triton X‐100 in PBS for 10 min at 4 °C and blocked with 4% bovine serum albumin (B2064, Sigma‐Aldrich, St. Louis, USA) in PBS for 0.5 h at 37 °C. The primary antibody was added and incubated with the cells at 4 °C overnight. At the end of incubation, the primary antibody was removed and the cells were washed with PBS. Then cells were incubated with Alexa Fluor 488‐ or Alexa Fluor 568‐conjugated secondary antibodies (A11008, A10037; 1:100, Thermo Fisher Scientific, Waltham, USA) at room temperature for 1 h. Nuclei were stained with DAPI for 5 min and then imaged with a fluorescence microscope (IX81‐FV1000, Olympus, Tokyo, Japan). The following primary antibodies were used: anti‐Bcl‐XL (ET1603‐28, Huabio, Hangzhou, China), anti‐USP13 (sc‐514416, Santa Cruz Biotechnology, Dallas, USA).

### Western Blot Analysis

Protein lysates (30–50 µg per sample) of cells or kidney tissues were preparing using lysis buffer (composed of 150 mm NaCl, 50 mm Tris‐HCl, 2 mm EGTA, 2 mm EDTA, 25 mm β‐sodium glycerophosphate, 25 mm NaF, 0.3% Triton X‐100, 0.3% NP‐40, 0.3% leupeptin, 0.1% NaVO_3_, and 0.1% PMSF). The protein lysates were separated on 8%, 10%, or 12% SDS‐PAGE gels, transferred to PVDF membranes (Millipore Corporation, Boston, USA) and blocked with a blocking buffer. Incubation of primary antibodies, secondary antibodies and the ECL‐Plus Kit (P2300, NCM Biotech, Suzhou, China) was performed to detect the bands.

Primary antibodies directed against cleaved PARP (ET1608‐10), Bcl‐XL (ET1603‐28), Bcl‐2 (ET1702‐53), BAK (ET1608‐21), BAX (ET1603‐34), ATG5 (ET1611‐38), NOTCH1 (ET1606‐55), SIGMAR1 (HA500511), γ‐H2AX (ET1602‐2), PRKN (ET1702‐60), USP13 (ET1706‐14), Phospho‐SMAD3 (S423/S425) (ET1609‐41) were purchased from Huabio (Hangzhou, China). Primary antibody against cleaved PARP (ab32064), α‐SMA (ab124964) were purchased from Abcam (Cambridge, UK). Primary antibody against ATG7 (2631s) and COL1A1 (84336S) were obtained from Cell Signaling Technology (Boston, USA). Primary antibody against LC3 (M152‐3) was purchased from MBL (Tokyo, Japan). Primary antibody against ACTB (db7283), FN1 (db2362), HA (db2603), SMAD3 (db1007) were obtained from Diagbio (Hangzhou, China). Primary antibody against MCL‐1 (A18001) was obtained from ABclonal Technology (Wuhan, China). Primary antibody against HSPA8 (sc‐7298), p53 (sc‐126) were purchased from Santa Cruz Biotechnology (Dallas, USA). Primary antibody against Myc (A00173) was obtained from GenScript Biotechnology (Nanjing, China). Anti‐Flag antibody (AYC01‐100) was purchased from Shanghai Yoche Biotechnology (Shanghai, China). HRP‐labeled secondary antibodies (GAR007, GAM007) were purchased from MultiSciences (Lianke) Biotech.

### Immunoprecipitation Assays

For the exogenous immunoprecipitation assay, cells were lysed in the 1% NP‐40 buffer (pH = 8.0): 25 mm Tris‐base, 150 mm NaCl, 10% glycerol, and 1% NP‐40; protease inhibitor cocktail (5871, Cell Signaling Technology, Boston, USA) added before use. The lysate (containing 500 µg of total protein) was centrifuged and immunoprecipitated with 20 µL anti‐Flag beads (SA042001, Smart‐Lifesciences, Changzhou, China) at 4 °C overnight. Then, the lysate was centrifuged to remove the supernatant and wash with 1% NP‐40 buffer, followed by Western blot analysis. For the endogenous immunoprecipitation assay, cells were lysed with the IP buffer (pH = 7.5): 50 mm Tris‐base, 150 mm NaCl, 2 mm EGTA, 25 mm NaF, and 25 mm β‐sodium glycerophosphate; protease inhibitor cocktail (5871, Cell Signaling Technology, Boston, USA). The lysate was centrifuged and immunoprecipitated with 20 µL protein A/G Plus‐agarose (sc‐2003, Santa Cruz Biotechnology, Dallas, USA) and a primary antibody (anti‐Bcl‐XL: ET1603‐28, Huabio, Hangzhou, China), IgG antibody (2729S, Cell Signaling Technology, Boston, USA) was used as a negative control. Co‐precipitated protein and initial whole‐cell lysates were analyzed by Western blot.

### qRT‐PCR

The procedures were described in a previous study.^[^ ^49^ ^]^ After treatment, cells were harvested with the Trizol reagent (15596‐026, Thermo Fisher Scientific, Waltham, USA). Equal amounts of RNA were reverse transcribed into complementary DNA with the cDNA reverse transcription kit (AT311, TransGen Biotech, Peking, China). qRT‐PCR was performed on a QuantStudio 3 real‐time PCR instrument (Applied Biosystems, Singapore) using the iTaq Universal SYBR Green Supermix (172‐5124, Bio‐Rad, Hercules, USA). The samples underwent two‐step amplification with an initial step at 95 °C (3 min), followed by 95 °C (3 s) and 60 °C (31 s) for 39 cycles. The melting curve was analyzed. Fold changes in the expression of each gene were calculated by the comparative threshold cycle (Ct) method using the formula 2^−(ΔΔCt)^. Two independent biological samples were quantified in technical duplicates and expression values were normalized to housekeeper gene.

The primer sequences were as follows:

Human‐*BCL2L1*‐Forward, 5′‐GGGAGCTGGTGGTTGACTTT‐3′;
Human‐*BCL2L1*‐Reverse, 5′‐CAGTGGCTCCATTCACCGC‐3′;
Mouse‐*α‐SMA*‐Forward, 5′‐GTGAAGAGGAAGACAGCACAG‐3′;
Mouse‐*α‐SMA* ‐Reverse, 5′‐GCCCATTCCAACCATTACTCC‐3′;
Mouse‐*Col1a1*‐Forward, 5′‐TGGACTTCCTGGTCCTCCTG‐3′;
Mouse‐*Col1a1*‐Reverse, 5′‐AGGCACGGAAACTCCAGC‐3′;
Mouse‐*Fn1*‐Forward, 5′‐TGCCTTCAACTTCTCCTGTG‐3′;
Mouse‐*Fn1*‐Reverse, 5′‐CACTAACCACGTACTCCACAG‐3′;
Mouse‐*Gapdh*‐Forward, 5′‐TCAACAGCAACTCCCACTCTTCCA‐3′;
Mouse‐*Gapdh*‐Reverse, 5′‐ACCCTGTTGCTGTAGCCGTATTCA‐3′;
Human‐*ACTB*‐Forward, 5′‐CACCATTGGCAATGAGCGGTTC‐3′;
Human*‐ACTB*‐Reverse, 5′‐AGGTCTTTGCGGATGTCCACGT‐3′.

### RNA Interference

Cells were seeded into 12‐well plates (5 × 10^4^ cells per well for HUVECs and 6 × 10^4^ cells per well for HK‐2) and grown to ≈50% confluence. Then, the cells were transfected with control siRNA (negative control) or siRNA against ATG5, ATG7, SIGMAR1 or NOTCH1 using siRNA Transfection Reagent (409‐10, Polyplus‐transfection, Illkirch, France) according to the manufacturer's instructions. The following siRNAs were purchased from GenePharma (Shanghai, China).

siRNA targeting ATG5, 5′‐CAUCUGAGCUACCCGGAUAdTdT‐3′;
siRNA targeting ATG7, 5′‐GCCGUGGAAUUGAUGGUAUUUdTdT‐3′;
siRNA targeting NOTCH1, 5′‐CCGGGACAUCACGGAUCAUAU‐3′;
siRNA targeting SIGMAR1, 5′‐GACUUCCUCACCCUCUUCUAU‐3′;
siRNA targeting USP13 #1, 5′‐CGACGAUUAUGAAUAUGAAGA‐3′;
siRNA targeting USP13 #2, 5′‐GCGACAGGGUCUACAAGAACG‐3′;
siRNA targeting negative control, 5′‐UUCUCCGAACGUGUCACGUdTdT‐3′.

### Plasmid Construction and Transfection

pCMV‐USP13 (P22551) was purchased from Miaolingbio (Wuhan, China) and pMD18‐T‐Bcl‐XL (HG10455‐M) was purchased from Sino Biological Inc (Beijing, China). cDNA was copied from these plasmids and change the vector with pcDNA3.0. pcDNA 3.0‐USP13‐Flag or pcDNA 3.0‐USP13‐Myc was generated by restriction digestion of the plasmids with BamHI plus XhoI, followed by ligations. pcDNA 3.0‐Bcl‐XL‐HA or pcDNA 3.0‐Bcl‐XL‐Flag was generated by restriction digestion of the plasmids with EcoRI plus XhoI, followed by ligations. EcoRI‐HF (R3101L), XhoI (R0146S), BamHI‐HF (R3136L) and T4 DNA ligase (M0202S) were purchased from New England Biolabs (Ipswich, USA). The USP13 enzyme inactive mutant USP13‐C345A was synthesized from wild‐type USP13 with a site‐directed mutagenesis kit (11003ES10, Yeasen Biotechnology, Shanghai, China). The primers used were listed as following: C345A F: 5′‐CAACAGCGCCTATCTCAGCTCTGTCATGCAGGC‐3′ and R: 5′‐AGATAGGCGCTGTTGCCCAGGTTCTTCAG‐3′. Cells were transfected with plasmids using the jetPRIME (101000046, Polyplus‐transfection, Illkirch, France) according to the manufacturer's instructions. Briefly, cells were seeded into specific dish or plate and grown to ≈70% confluence. HEK293T cells were seeded in 6 cm culture dish (1.2 × 10^6^ cells per dish), HK‐2 cells and HUVECs were seeded in 12‐well plates (8 × 10^4^ cells per well for HK‐2 and 6.5 × 10^4^ cells per well for HUVECs). Then, the cells were transfected with transfection reagent. The ratio of plasmid and reagent is 1 µg:2 µL. After 4 h‐transfection, the medium was replaced with fresh complete medium. For 6 cm culture dish, total 3 µg plasmids were used and the amount was divided equally for each plasmid. For 12‐well plate, total 0.2–0.4 µg plasmids were used.

### RNA‐Seq Analysis

Total RNAs were isolated from HUVECs of control, nilo‐, CQ‐, nilo plus CQ‐treated using TRIzol reagent according to the manufacturer's protocol. An RNA‐seq analysis was outsourced to Applied Protein Technology (Shanghai, China). The RNA‐seq data were deposited in the NCBI Gene Expression Omnibus database (GSE212122).

### COMET Assay

The COMET assay was performed as described previously.^[^ ^50^ ^]^ First, a single‐cell suspension was prepared at a density of ≈2 × 10^4^ cells mL^−1^ in PBS. Next, preheated 0.5% normal‐gelling‐temperature agarose was placed onto a microscope slide. Then, 85 µL 0.5% low‐gelling‐temperature agarose with ≈2000 cells and 0.5% low‐gelling temperature agarose were spread successively (placed for over 10 min in turn). The slides were submerged into alkaline solution (10% DMSO, 1% Triton X‐100 and 89% lysis buffer containing 2.5 m NaCl, 100 mm Na_2_EDTA, and 10 mm Tris at pH = 10) for 1 h at 4 °C to lyse the samples. The slides were dipped in a horizontal electrophoresis chamber filled with cold alkaline solution (pH = 12.3) for 20 min to unravel DNA, and electrophoresis was performed for 20 min at 300 mA. Subsequently, the samples were neutralized with Tris‐HCl (pH = 7.5) for 15 min and then dehydrated in 70% ethanol. Finally, the samples were stained with DAPI for 5 min, and imaged by fluorescence microscopy.

### Bioinformatics Analysis

The predicted human E3 ubiquitination ligase‐Bcl‐XL interaction and deubiquitinase‐Bcl‐XL interaction were analyzed using UbiBrowser. The predict outcomes were processed for the intuitive display.

### Statistics

Data are represented as the mean ± standard deviation (SD). Statistical comparisons between two groups were performed using a 2‐tailed Student's *t*‐test; comparisons between more than two groups were made using a one‐way analysis of variance (ANOVA) with follow‐up Dunnett T3 tests. *P* value of <0.05 was considered statistically significant. Statistical analysis was performed using GraphPad Prism Version 8.0. All the statistical details of the experiments can be found in the figure legends.

## Conflict of Interest

The authors declare no conflict of interest.

## Supporting information

Supporting Information

## Data Availability

The data that support the findings of this study are openly available in NCBI GEO at https://www.ncbi.nlm.nih.gov/geo/query/acc.cgi?acc=GSE212122, reference number 212122.

## References

[1] a) H. M. Kantarjian , T. P. Hughes , R. A. Larson , D. W. Kim , S. Issaragrisil , P. le Coutre , G. Etienne , C. Boquimpani , R. Pasquini , R. E. Clark , V. Dubruille , I. W. Flinn , S. Kyrcz‐Krzemien , E. Medras , M. Zanichelli , I. Bendit , S. Cacciatore , K. Titorenko , P. Aimone , G. Saglio , A. Hochhaus , Leukemia 2021, 35, 440; 33414482 10.1038/s41375-020-01111-2 PMC7862065

[2] K. Sasaki , A. Lahoti , E. Jabbour , P. Jain , S. Pierce , G. Borthakur , N. Daver , T. Kadia , N. Pemmaraju , A. Ferrajoli , S. O'Brien , H. Kantarjian , J. Cortes , Clin. Lymphoma Myeloma Leuk. 2016, 16, 152. 26796981 10.1016/j.clml.2015.12.003 PMC4769134

[3] a) T. A. Fleisher , Ann. Allergy Asthma Immunol. 1997, 78, 245; 9087147 10.1016/S1081-1206(10)63176-6

[4] a) N. Pabla , Z. Dong , Kidney Int. 2008, 73, 994; 18272962 10.1038/sj.ki.5002786

[5] R. Singh , A. Letai , K. Sarosiek , Nat. Rev. Mol. Cell Biol. 2019, 20, 175. 30655609 10.1038/s41580-018-0089-8 PMC7325303

[6] a) R. F. Schwabe , T. Luedde , Nat. Rev. Gastroenterol. Hepatol. 2018, 15, 738; 30250076 10.1038/s41575-018-0065-y PMC6490680

[7] a) W. A. Siddiqui , A. Ahad , H. Ahsan , Arch. Toxicol. 2015, 89, 289; 25618543 10.1007/s00204-014-1448-7

[8] T. Moldoveanu , P. E. Czabotar , Cold Spring Harb. Perspect Biol. 2020, 12, a036319. 31570337 10.1101/cshperspect.a036319 PMC7111251

[9] F. Edlich , Biochem. Biophys. Res. Commun. 2018, 500, 26. 28676391 10.1016/j.bbrc.2017.06.190

[10] C. F. A. Warren , M. W. Wong‐Brown , N. A. Bowden , Cell Death Dis. 2019, 10, 177. 30792387 10.1038/s41419-019-1407-6 PMC6384907

[11] G. Dong , L. Wang , C. Y. Wang , T. Yang , M. V. Kumar , Z. Dong , J. Pharmacol. Exp. Ther. 2008, 325, 978. 18310471 10.1124/jpet.108.137398

[12] X. L. Xiong , R. H. Jia , D. P. Yang , G. H. Ding , Pharmacol. Res. 2006, 54, 253. 16828304 10.1016/j.phrs.2006.05.005

[13] a) D. Chen , F. Gao , B. Li , H. Wang , Y. Xu , C. Zhu , G. Wang , J. Biol. Chem. 2010, 285, 38214; 20889974 10.1074/jbc.M110.101469 PMC2992255

[14] a) D. Popovic , D. Vucic , I. Dikic , Nat. Med. 2014, 20, 1242; 25375928 10.1038/nm.3739

[15] D. Mennerich , K. Kubaichuk , T. Kietzmann , Trends Cancer 2019, 5, 632. 31706510 10.1016/j.trecan.2019.08.005

[16] J. A. Harrigan , X. Jacq , N. M. Martin , S. P. Jackson , Nat. Rev. Drug Discov. 2018, 17, 57. 28959952 10.1038/nrd.2017.152 PMC7097658

[17] J. Liu , H. Xia , M. Kim , L. Xu , Y. Li , L. Zhang , Y. Cai , H. V. Norberg , T. Zhang , T. Furuya , M. Jin , Z. Zhu , H. Wang , J. Yu , Y. Li , Y. Hao , A. Choi , H. Ke , D. Ma , J. Yuan , Cell 2011, 147, 223. 21962518 10.1016/j.cell.2011.08.037 PMC3441147

[18] a) S. Zhang , M. Zhang , Y. Jing , X. Yin , P. Ma , Z. Zhang , X. Wang , W. Di , G. Zhuang , Nat. Commun. 2018, 9, 215; 29335437 10.1038/s41467-017-02693-9 PMC5768685

[19] H. Xie , J. Zhou , X. Liu , Y. Xu , A. J. Hepperla , J. M. Simon , T. Wang , H. Yao , C. Liao , A. S. Baldwin , K. Gong , Q. Zhang , Proc. Natl. Acad. Sci. USA 2022, 119, e2119854119. 36037364 10.1073/pnas.2119854119 PMC9457248

[20] a) A. Jorge , C. Ung , L. H. Young , R. B. Melles , H. K. Choi , Nat. Rev. Rheumatol. 2018, 14, 693; 30401979 10.1038/s41584-018-0111-8

[21] a) J. Malhotra , S. Jabbour , M. Orlick , G. Riedlinger , Y. Guo , E. White , J. Aisner , Cancer Treat. Res. Commun. 2019, 21, 100158; 31521049 10.1016/j.ctarc.2019.100158

[22] a) H. Maes , A. Kuchnio , A. Peric , S. Moens , K. Nys , K. De Bock , A. Quaegebeur , S. Schoors , M. Georgiadou , J. Wouters , S. Vinckier , H. Vankelecom , M. Garmyn , A. C. Vion , F. Radtke , C. Boulanger , H. Gerhardt , E. Dejana , M. Dewerchin , B. Ghesquiere , W. Annaert , P. Agostinis , P. Carmeliet , Cancer Cell 2014, 26, 190; 25117709 10.1016/j.ccr.2014.06.025

[23] D. E. Spiers , V. Candas , J. Appl. Physiol. Respir. Environ. Exerc. Physiol. 1984, 56, 240. 6693327 10.1152/jappl.1984.56.1.240

[24] Y. Seki , O. Nagano , R. Koda , S. Morita , G. Hasegawa , Int. J. Hematol. 2020, 112, 584. 32557125 10.1007/s12185-020-02913-x

[25] a) W. K. Han , V. Bailly , R. Abichandani , R. Thadhani , J. V. Bonventre , Kidney Int. 2002, 62, 237; 12081583 10.1046/j.1523-1755.2002.00433.x

[26] a) S. E. Thomas , T. F. Andoh , R. H. Pichler , S. J. Shankland , W. G. Couser , W. M. Bennett , R. J. Johnson , Kidney Int. 1998, 53, 897; 9551396 10.1111/j.1523-1755.1998.00835.x

[27] a) X. Chen , H. Tan , J. Xu , Y. Tian , Q. Yuan , Y. Zuo , Q. Chen , X. Hong , H. Fu , F. F. Hou , L. Zhou , Y. Liu , Kidney Int. 2022, 102, 506; 35644285 10.1016/j.kint.2022.04.028

[28] J. Zhang , A. Salminen , X. Yang , Y. Luo , Q. Wu , M. White , J. Greenhaw , L. Ren , M. Bryant , W. Salminen , T. Papoian , W. Mattes , Q. Shi , Arch. Toxicol. 2017, 91, 2921. 28032146 10.1007/s00204-016-1918-1 PMC5515969

[29] a) F. J. Bock , S. W. G. Tait , Nat. Rev. Mol. Cell Biol. 2020, 21, 85; 31636403 10.1038/s41580-019-0173-8

[30] A. Perelman , C. Wachtel , M. Cohen , S. Haupt , H. Shapiro , A. Tzur , Cell Death Dis. 2012, 3, e430. 23171850 10.1038/cddis.2012.171 PMC3542606

[31] a) F. Ni , C. Y. Yan , S. Zhou , P. Y. Hui , Y. H. Du , L. Zheng , J. Yu , X. J. Hu , Z. G. Zhang , Cancer Chemother. Pharmacol. 2018, 82, 593; 30032449 10.1007/s00280-018-3651-3

[32] M. Mauthe , I. Orhon , C. Rocchi , X. Zhou , M. Luhr , K. J. Hijlkema , R. P. Coppes , N. Engedal , M. Mari , F. Reggiori , Autophagy 2018, 14, 1435. 29940786 10.1080/15548627.2018.1474314 PMC6103682

[33] Y. Li , P. Xie , L. Lu , J. Wang , L. Diao , Z. Liu , F. Guo , Y. He , Y. Liu , Q. Huang , H. Liang , D. Li , F. He , Nat. Commun. 2017, 8, 347. 28839186 10.1038/s41467-017-00299-9 PMC5570908

[34] I. Compter , D. B. P. Eekers , A. Hoeben , K. M. A. Rouschop , B. Reymen , L. Ackermans , J. Beckervordersantforth , N. J. C. Bauer , M. M. Anten , P. Wesseling , A. A. Postma , D. De Ruysscher , P. Lambin , Autophagy 2021, 17, 2604. 32866424 10.1080/15548627.2020.1816343 PMC8496728

[35] J. M. Adams , S. Cory , Science 1998, 281, 1322. 9735050 10.1126/science.281.5381.1322

[36] a) M. El Mouedden , G. Laurent , M. P. Mingeot‐Leclercq , P. M. Tulkens , Toxicol. Sci. 2000, 56, 229; 10869472 10.1093/toxsci/56.1.229

[37] F. Edlich , S. Banerjee , M. Suzuki , M. M. Cleland , D. Arnoult , C. Wang , A. Neutzner , N. Tjandra , R. J. Youle , Cell 2011, 145, 104. 21458670 10.1016/j.cell.2011.02.034 PMC3070914

[38] a) C. O. Eno , E. F. Eckenrode , K. E. Olberding , G. Zhao , C. White , C. Li , Mol. Biol. Cell 2012, 23, 2605; 22573883 10.1091/mbc.E12-02-0090 PMC3386223

[39] a) C. Tang , M. J. Livingston , R. Safirstein , Z. Dong , Nat. Rev. Nephrol. 2023, 19, 53; 36229672 10.1038/s41581-022-00631-7

[40] a) I. Burlaka , L. M. Nilsson , L. Scott , U. Holtback , A. C. Eklof , A. B. Fogo , H. Brismar , A. Aperia , Kidney Int. 2016, 90, 135; 27217195 10.1016/j.kint.2016.03.026 PMC6101029

[41] C. Han , L. Yang , H. H. Choi , J. Baddour , A. Achreja , Y. Liu , Y. Li , J. Li , G. Wan , C. Huang , G. Ji , X. Zhang , D. Nagrath , X. Lu , Nat. Commun. 2016, 7, 13525. 27892457 10.1038/ncomms13525 PMC5133706

[42] a) I. Ben‐Zvi , S. Kivity , P. Langevitz , Y. Shoenfeld , Clin. Rev. Allergy Immunol. 2012, 42, 145; 21221847 10.1007/s12016-010-8243-x PMC7091063

[43] a) F. L. Pagan , M. L. Hebron , B. Wilmarth , Y. Torres‐Yaghi , A. Lawler , E. E. Mundel , N. Yusuf , N. J. Starr , M. Anjum , J. Arellano , H. H. Howard , W. Shi , S. Mulki , T. Kurd‐Misto , S. Matar , X. Liu , J. Ahn , C. Moussa , JAMA Neurol. 2020, 77, 309; 31841599 10.1001/jamaneurol.2019.4200 PMC6990742

[44] L. Levato , R. Cantaffa , M. G. Kropp , D. Magro , E. Piro , S. Molica , Eur. J. Haematol. 2013, 90, 531. 23506097 10.1111/ejh.12096

[45] M. Cellier , D. Bourneau‐Martin , C. Abbara , A. Crosnier , L. Lagarce , A. S. Garnier , M. Briet , Cancers 2023, 15, 2041. 37046701 10.3390/cancers15072041 PMC10093506

[46] a) M. S. Zaghloul , R. S. Abdelrahman , Toxicology 2019, 427, 152303; 31593741 10.1016/j.tox.2019.152303

[47] S. Tugues , G. Fernandez‐Varo , J. Munoz‐Luque , J. Ros , V. Arroyo , J. Rodes , S. L. Friedman , P. Carmeliet , W. Jimenez , M. Morales‐Ruiz , Hepatology 2007, 46, 1919. 17935226 10.1002/hep.21921

[48] A. Kazama , A. Katagiri , S. Ishikawa , T. Mizusawa , BMJ Case Rep. 2017, 2017. 10.1136/bcr-2017-221494 PMC5624229 28851716

[49] P. Luo , H. Yan , J. Du , X. Chen , J. Shao , Y. Zhang , Z. Xu , Y. Jin , N. Lin , B. Yang , Q. He , Autophagy 2021, 17, 3221. 33315519 10.1080/15548627.2020.1851492 PMC8526032

[50] L. Jiang , Y. Zeng , L. Ai , H. Yan , X. Yang , P. Luo , B. Yang , Z. Xu , Q. He , Biochem. Pharmacol. 2022, 201, 115105. 35617997 10.1016/j.bcp.2022.115105

