# Supplementary material for: Chloroquine Intervenes Nephrotoxicity of Nilotinib through Deubiquitinase USP13‐Mediated Stabilization of Bcl‐XL
Source: Adv Sci (Weinh). 2023 Jul 14;10(26):2302002. doi: 10.1002/advs.202302002 (PMC10502815; doi:10.1002/advs.202302002)
Supplement: Supplementary file 1 — Supporting Information [file ADVS-10-2302002-s001.pdf]

ADVANCED SCIENCE
Open Access

Supporting Information

for *Adv. Sci.*, DOI 10.1002/advs.202302002

Chloroquine Intervenes Nephrotoxicity of Nilotinib through Deubiquitinase USP13-Mediated Stabilization of Bcl-XL

*Hao Yan, Xiangliang Huang, Jiangxin Xu, Ying Zhang, Jiajia Chen, Zhifei Xu, Hui Li, Zeng Wang, Xiaochun Yang, Bo Yang, Qiaojun He and Peihua Luo**

Chloroquine Intervenes Nephrotoxicity of Nilotinib Through Deubiquintinase USP13-Mediated Stabilization of Bcl-XL

Hao Yan, PhD [1], Xiangliang Huang, BS [1], Jiangxin Xu, BS [1], Ying Zhang, MM [1], Jiajia Chen, BS [1], Zhifei Xu, PhD [1], Hui Li, PhD [1], Zeng Wang, PhD [2], Xiaochun Yang, PhD [1], Bo Yang, PhD [3], Qiaojun He, PhD [1,4], Peihua Luo, PhD [1,5*]

[1]Center for Drug Safety Evaluation and Research of Zhejiang University, College of Pharmaceutical Sciences, Zhejiang University, Hangzhou 310058, China

[2]Department of Pharmacy, Zhejiang Cancer Hospital, Hangzhou 310005, China

[3]Institute of Pharmacology & Toxicology, College of Pharmaceutical Sciences, Zhejiang University, Hangzhou 310058, China

[4]Innovation Institute for Artificial Intelligence in Medicine of Zhejiang University, Hangzhou 310018, China

[5]Department of Cardiology, Second Affiliated Hospital, School of Medicine, Zhejiang University, Hangzhou 310009, China

Supporting information includes 18 Supplementary Figures and legends.

Supplementary Figures and legends

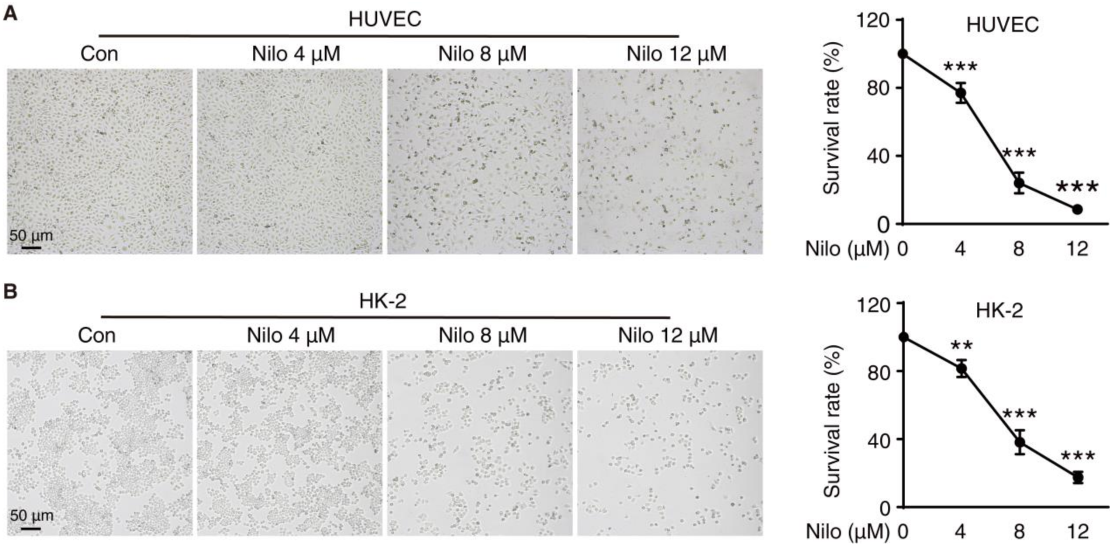

Supplemental Figure 1. The morphology and survival rate change of HUVECs and HK-2 cells after treatment with nilotinib. (A and B) HUVECs and HK-2 cells were treated with 0, 4, 8, 12 μM nilotinib for 24 h. The morphology of cells was observed under light microscope. Scale bar = 50 μm. Cell survival rate was measured by SRB assay. $n = 3$ independent experiments. The results are presented as the mean ± SD. The P value was calculated by one-way ANOVA (Dunnett's multiple comparisons test). $^{**}P < 0.01$; $^{***}P < 0.001$.

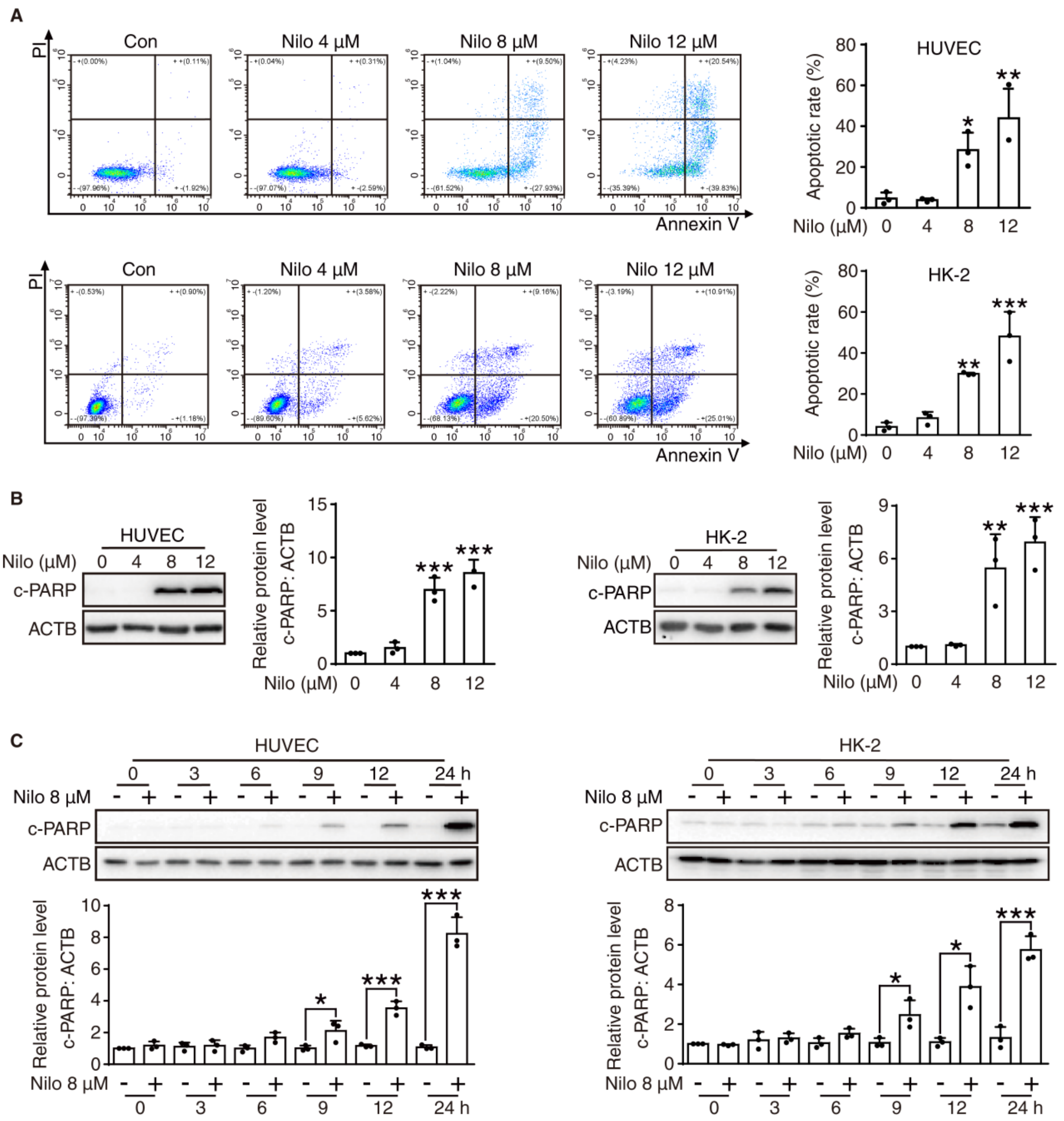

Supplemental Figure 2. Nilotinib induces cell apoptosis in HUVECs and HK2 cells. **(A)** HUVECs or HK-2 cells were treated with 0, 4, 8, 12 μM nilotinib for 24 h. The apoptotic rate was analyzed by flow cytometry combination with Annexin V/PI staining and representative images are shown. *n* = 3 independent experiments. **(B and C)** HUVECs or HK-2 cells were treated with 0, 4, 8 ,12 μM nilotinib for 24 h or 8 μM nilotinib for 0, 3, 6, 9, 12, 24 h. *n* = 3 independent experiments. Relative expression of c-PARP was analyzed by western blot with ACTB as a loading control. The results are presented as the mean ± SD. The *P* value was calculated by one-way ANOVA (Dunnett's multiple comparisons test). *$P < 0.05$; ***$P < 0.001$.

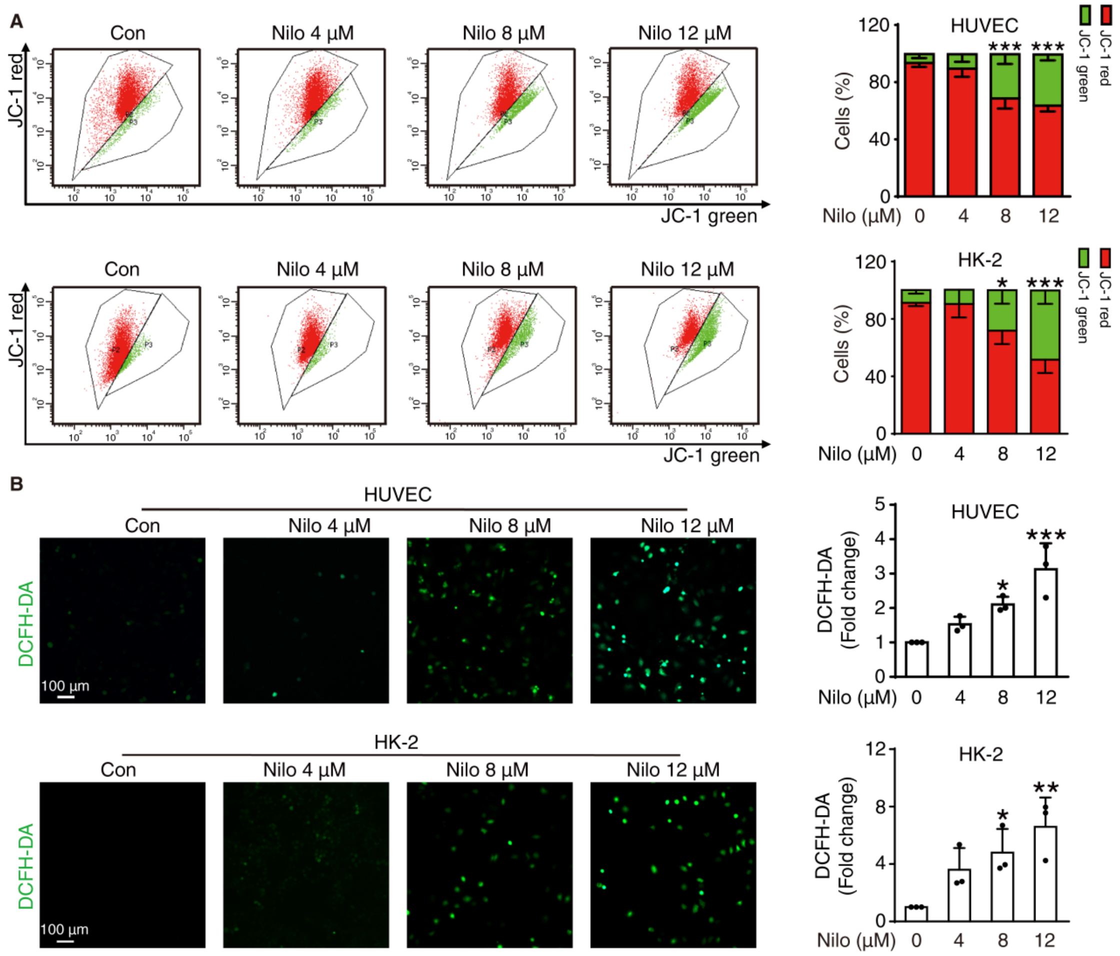

Supplemental Figure 3. Nilotinib induces mitochondrial dysfunction and ROS accumulation in HUVECs and HK-2 cells. (A and B) HK-2 cells were treated with 0, 4, 8 ,12 μM nilotinib for 24 h. *n* = 3 independent experiments. **(A)** Flow cytometry combination with JC-1 staining was used to measure the mitochondrial membrane potential, and representative images are shown. **(B)** DCFH-DA probes staining was used to detect the level of ROS. Representative fluorescent images of intracellular ROS are shown. Scale bar = 100 μm. Flow cytometry was applied to quantify the relative intracellular ROS level. The results are presented as the mean ± SD. The *P* value was calculated by one-way ANOVA (Dunnett's multiple comparisons test). **P* < 0.05; ***P* < 0.01; ****P* < 0.001.

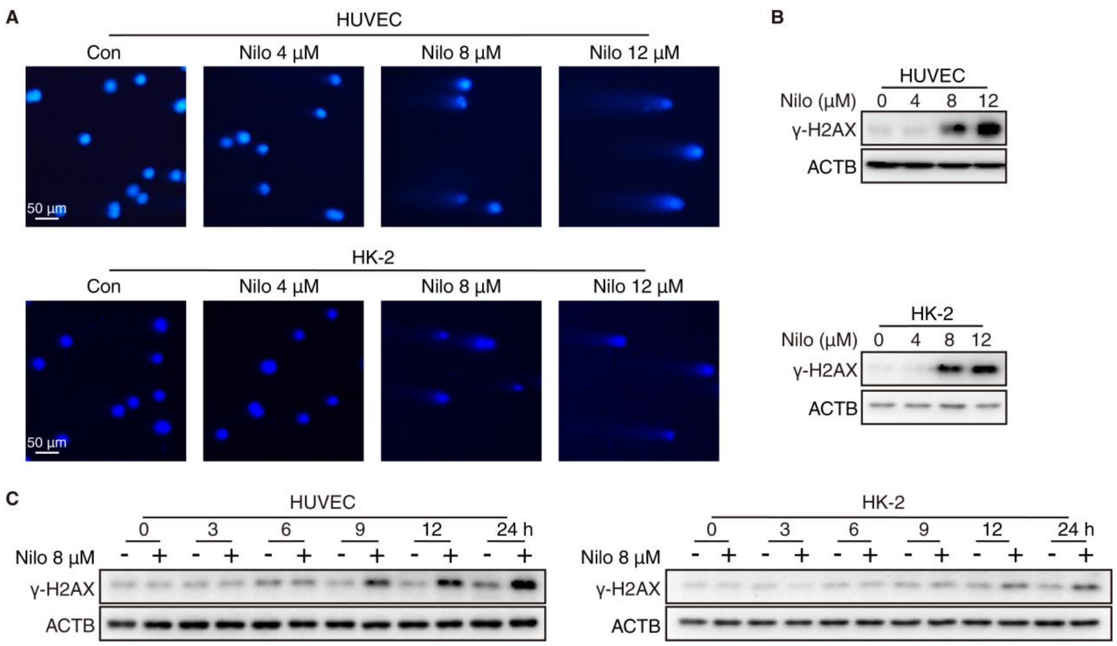

Supplemental Figure 4. Nilotinib induces DNA damage in kidney cells. (A-C) HUVECs and HK-2 cells were treated with 0, 4, 8, 12 μM nilotinib for 24 h. COMET assay was introduced to detect the DNA double strands break. **(A)** Representative fluorescent images of HUVECs and HK-2 cells are shown. Scale bar = 50 μm. **(B)** Relative expression of γ-H2AX was analyzed by western blot in HUVECs and HK-2 cells. **(C)** HUVECs and HK-2 cells were treated with 8 μM nilotinib for 0, 3, 6, 9, 12, 24 h. Relative expression of γ-H2AX was analyzed by western blot. ACTB was used as a loading control.

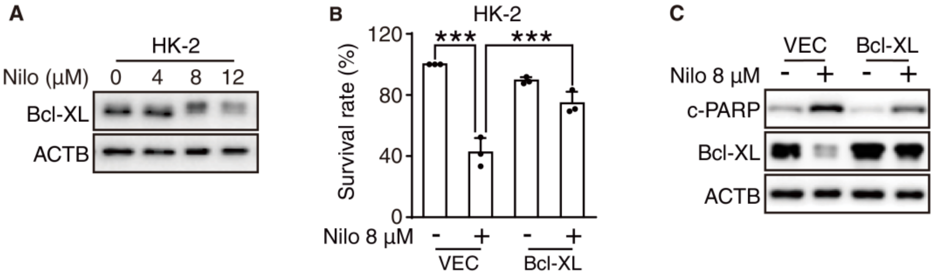

Supplemental Figure 5. Nilotinib induces cell apoptosis via reducing Bcl-XL in HK-2 cells. (A) HK-2 cells were treated with 0, 4, 8, 12 μM nilotinib for 24 h. Relative expression of Bcl-XL was analyzed by western blot with ACTB as a loading control. **(B and C)** HK-2 cells were transfected with pcDNA3.0-Bcl-XL plasmid or vector, followed by treatment with or without nilotinib for 24 h. **(B)** The cell survival rate was

measured by SRB assay. n = 3 independent experiments. **(C)** The relative expression levels of c-PARP and Bcl-XL were determined by western blot with ACTB as a loading control. The results are presented as the mean ± SD. The P value was calculated by one-way ANOVA (Dunnett's multiple comparisons test). ***P < 0.001.

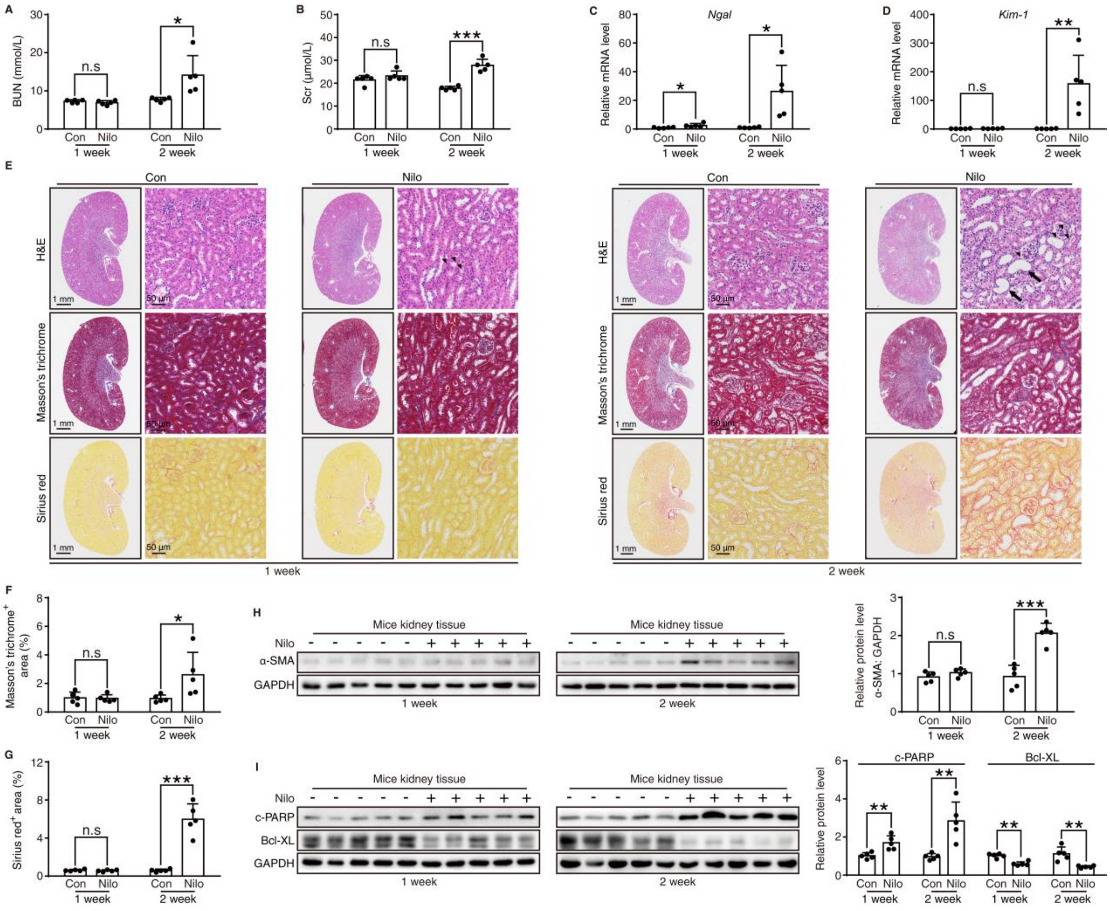

Supplemental Figure 6. The pathologic changes of nilotinib-induced nephrotoxicity *in vivo.* **(A-I)** C57BL/6J mice were administered 0.5% CMC-Na or nilotinib (300 mg/kg) for 1 week and 2 weeks (n = 5 per group). Kidneys and serum were harvested. **(A)** BUN and **(B)** Scr levels were analyzed. **(C and D)** The mRNA expression of *Ngal* and *Kim-1* were analyzed by qRT-PCR. **(E)** Representative panoramic images and local zoomed images of kidney tissues with hematoxylin and eosin staining (H&E), Masson's trichrome and Sirius red staining, Scale bar = 1 mm or 50 μm, respectively. Black arrowheads indicated the apoptosis body. Black arrows indicated the degeneration and expansion of renal tubular. Quantitative analysis was performed to detect Masson's trichrome positive area **(F)** and Sirius red staining

positive area **(G)**. **(H)** Relative expression of α-SMA was analyzed by western blot. **(I)** Relative expression levels of c-PARP and Bcl-XL were analyzed by western blot. GAPDH was used as a loading control. The results are presented as the mean ± SD. The *P* value was calculated by Student's *t* test (unpaired, two-tailed, 2 groups). n.s. = no significance; $*P < 0.05$; $**P < 0.01$; $***P < 0.001$.

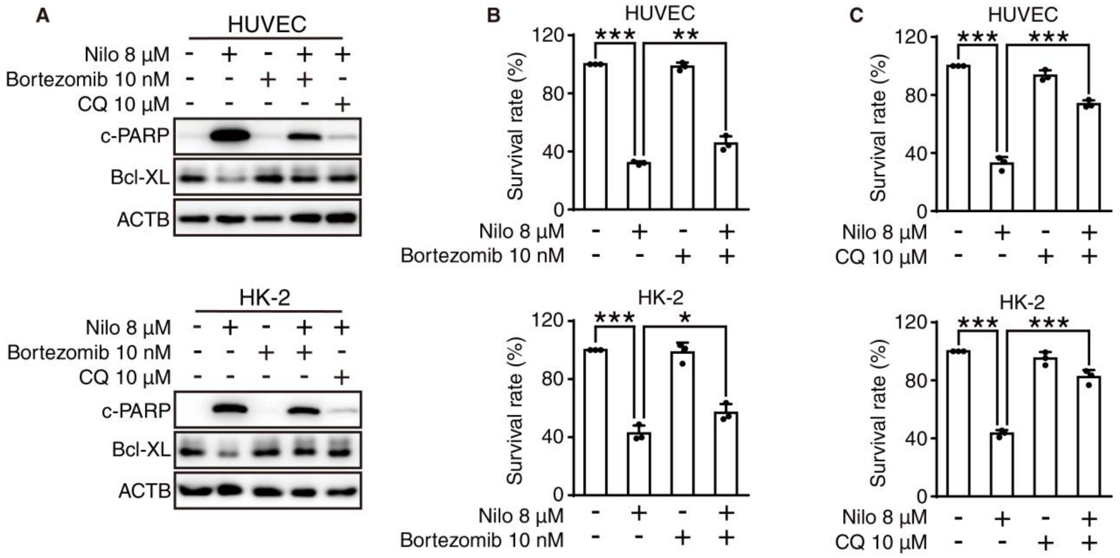

Supplemental Figure 7. The effect of Bortezomib and CQ on nilotinib-induced Bcl-XL reduction and cell survival. (A) HUVECs or HK-2 cells were treated with nilotinib with or without 10 nM Bortezomib, 10 μM CQ for 24 h. Relative expression of c-PARP and Bcl-XL was analyzed by western blot. ACTB was used as a loading control. **(B)** HUVECs or HK-2 cells were treated with nilotinib with or without 10 nM Bortezomib for 24 h. Cell survival rate was measured by SRB assay. **(C)** HUVECs or HK-2 cells were treated with nilotinib with or without 10 μM CQ for 24 h. Cell survival rate was measured by SRB assay. The *P* value was calculated by one-way ANOVA (Dunnett's multiple comparisons test). $*P < 0.05$; $**P < 0.01$; $***P < 0.001$.

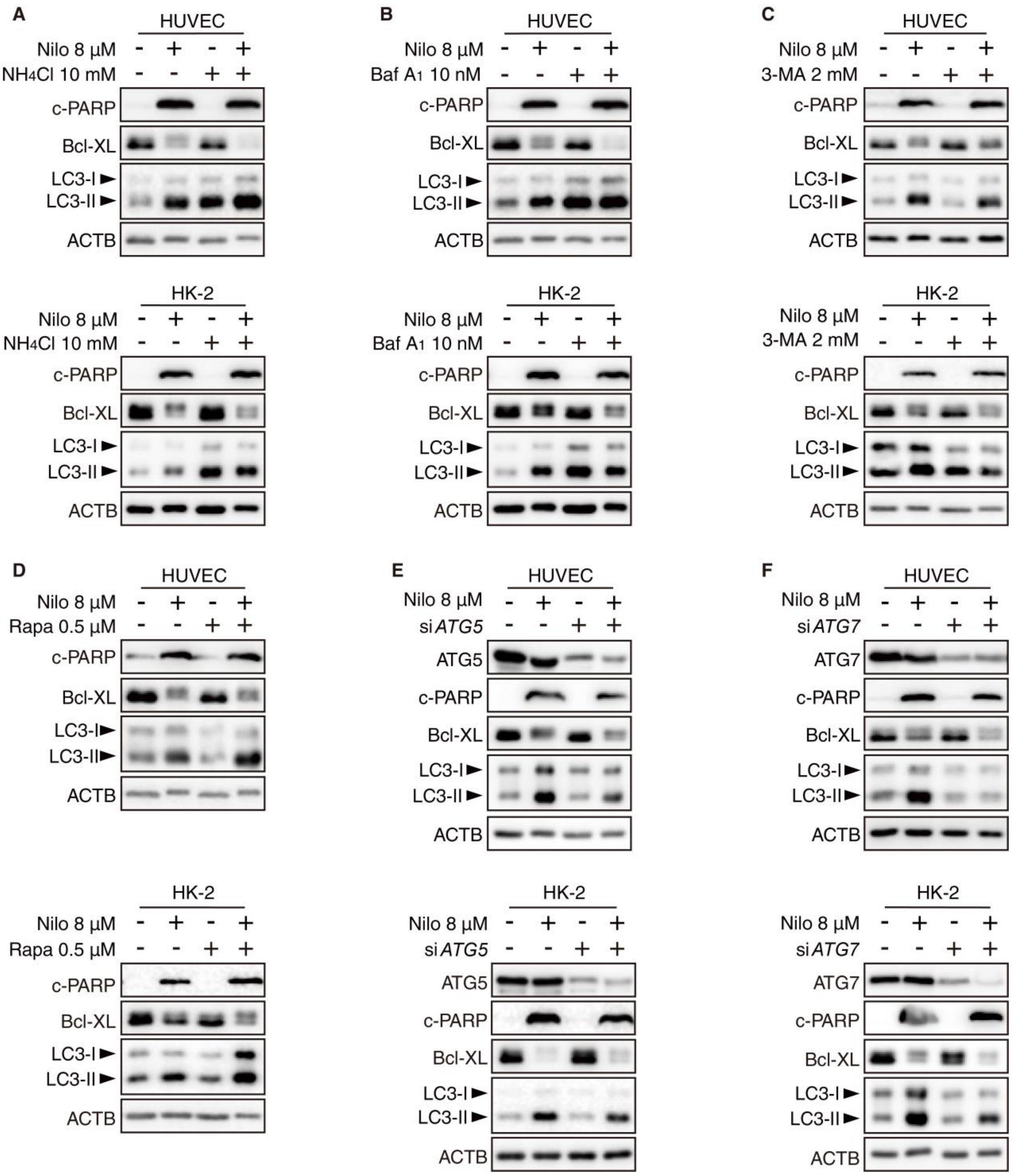

Supplemental Figure 8. CQ restores nilotinib-induced reduction of Bcl-XL independently of autophagy inhibition in kidney cells. HUVECs and HK-2 cells were treated with **(A)** 10 mM NH_4Cl or **(B)** 10 nM Bafilomycin A_1 or **(C)** 2 mM 3-MA or **(D)** 0.5 μM Rapamycin, followed by treatment with or without nilotinib for 24 h. Expression of c-PARP, Bcl-XL and LC3 was analyzed by western blot with ACTB as a loading control. HUVECs and HK-2 cells were transfected with siRNA, **(E)** targeting ATG5 (si*ATG5*) or **(F)** ATG7 (si*ATG7*), or non-targeting siRNA for 24 h as indicated, followed by treatment with or without nilotinib. Relative protein expression was analyzed by western blot with ACTB as a loading control.

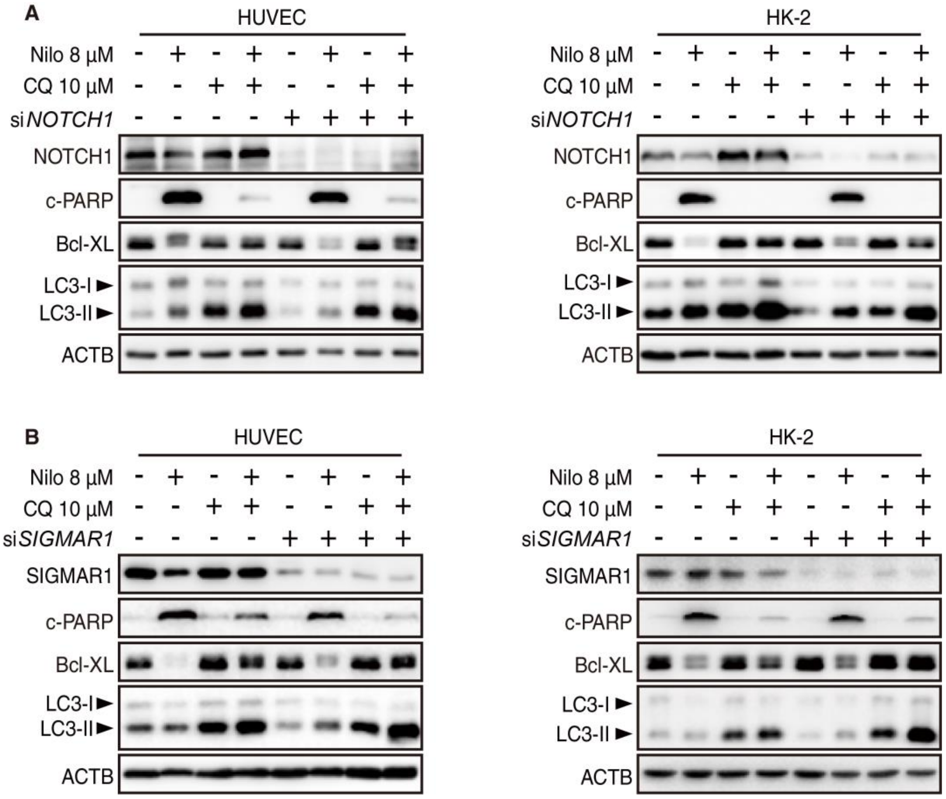

Supplemental Figure 9. CQ restores nilotinib-induced reduction of Bcl-XL independently of known non-autophagy dependent targets. (A and B) HUVECs and HK-2 cells were transfected with siRNA, targeting NOTCH1 (si*NOTCH1*) or SIGMAR1 (si*SIGMAR1)*, or non-targeting siRNA for 24 h as indicated, followed by treatment with or without nilotinib. Relative protein expression was analyzed by western blot with ACTB as a loading control.

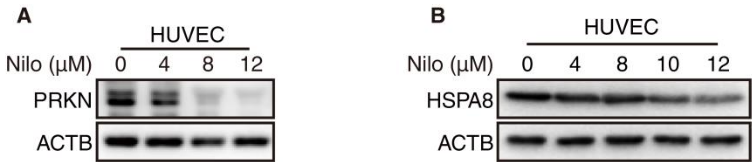

Supplemental Figure 10. PRKN and HSPA8 were down-regulated after nilotinib's treatment. (A and B) HUVECs were treated with different concentration of nilotinib as indicated for 24 h. Relative expression of **(A)** PRKN or **(B)** HSPA8 was analyzed by western blot with ACTB as a loading control.

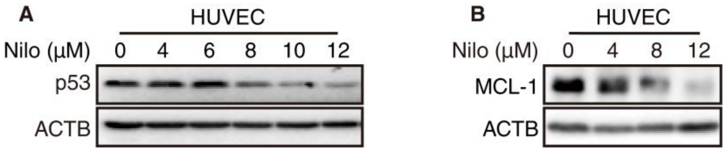

Supplemental Figure 11. p53 and MCL-1 were reduced after nilotinib's treatment. (A and B) HUVECs were treated with different concentration of nilotinib as indicated for 24 h. Relative expression of **(A)** p53 or **(B)** MCL-1 was analyzed by western blot with ACTB as a loading control.

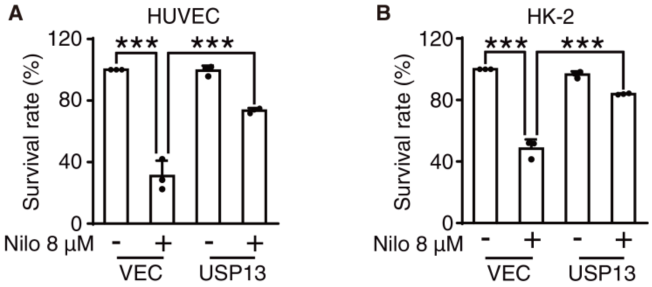

Supplemental Figure 12. Overexpression of USP13 reversed nilotinib-induced kidney cells death. (A and B) HUVECs and HK-2 cells were transfected with pcDNA3.0-USP13 for 24 h, followed by the treatment of 8 μM nilotinib, and cell survival rate was measured by SRB assay. $n = 3$ independent experiments. The results are presented as the mean ± SD. The P value was calculated by one-way ANOVA (Dunnett's multiple comparisons test). ***$P < 0.001$.

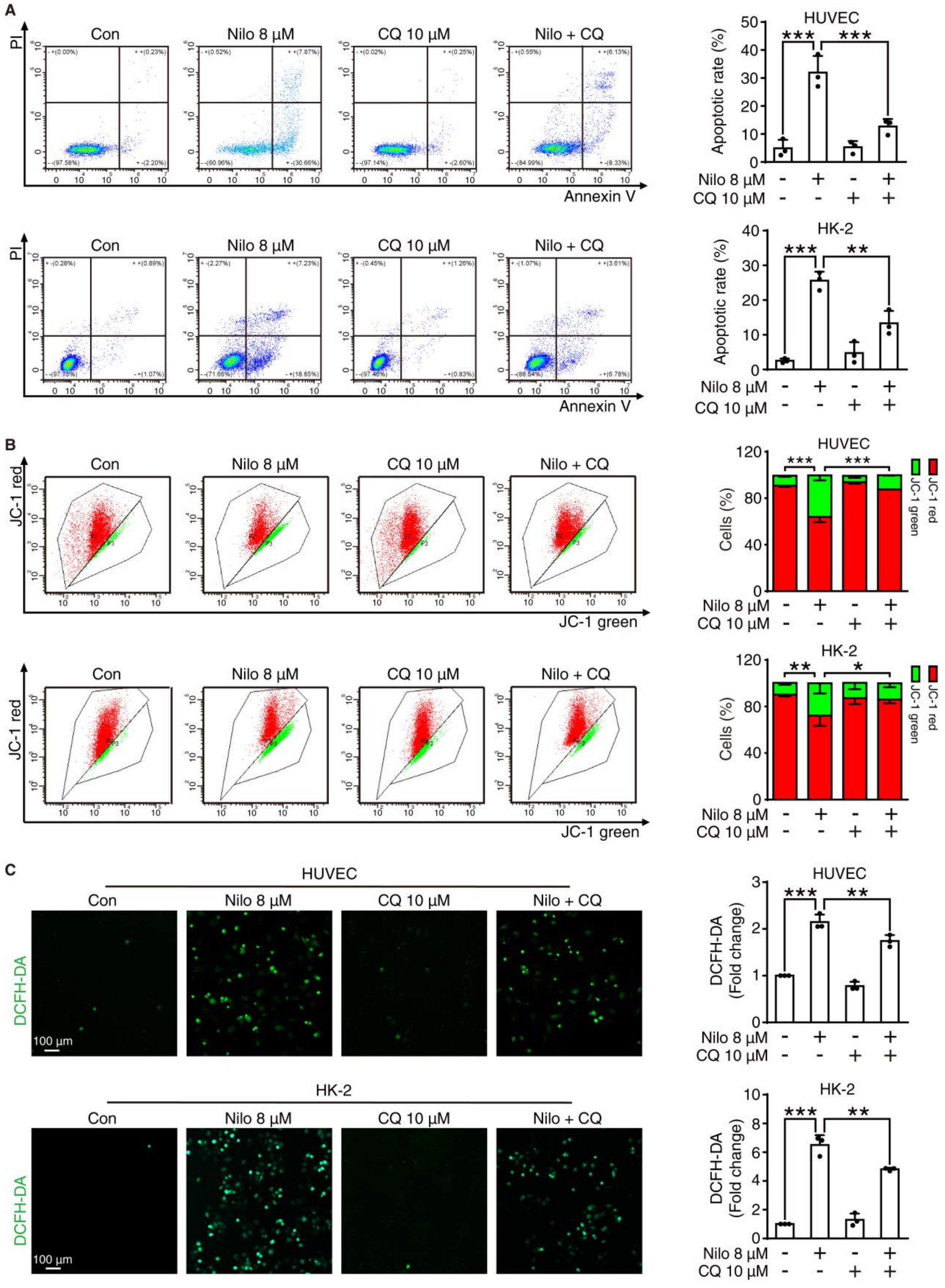

Supplemental Figure 13. CQ relieves nilotinib-induced nephrotoxicity *in vitro*. (A-C) HUVECs and HK-2 cells were treated with 8 μM nilotinib with or without 10 μM CQ for 24 h as indicated. *n* = 3 independent experiments. **(A)** The apoptotic rate was analyzed by flow cytometry combination with Annexin V/PI staining and representative images are shown. **(B)** The loss of mitochondrial membrane potential was detected by

flow cytometry combination with JC-1 staining and representative images are shown. **(C)** Representative fluorescent images of intracellular ROS are shown. Scale bar = 100 μm. The quantitative data of intracellular ROS level was analyzed by flow cytometry. The results are presented as the mean ± SD. The *P* value was calculated by one-way ANOVA (Dunnett's multiple comparisons test). n.s = no significance; $^*P < 0.05$; $^{**}P < 0.01$; $^{***}P < 0.001$.

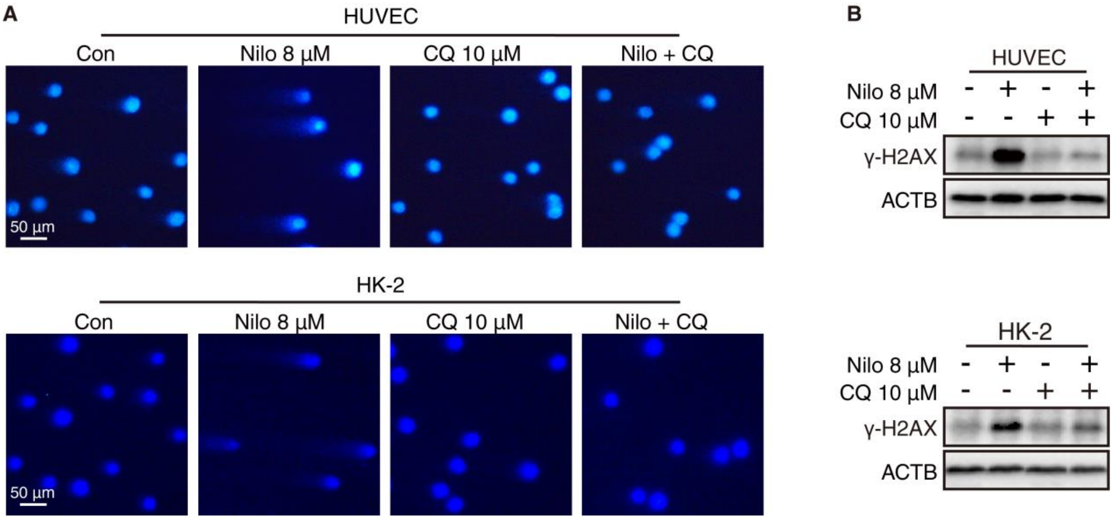

Supplemental Figure 14. CQ rescued nilotinib-induced DNA damage. (A and B) HUVECs and HK-2 cells were treated with 8 μM nilotinib with or without 10 μM CQ for 24 h. COMET assay was introduced to detect the DNA double strands break. **(A)** Representative images of HUVECs and HK-2 cells are shown. Scale bar = 50 μm. **(B)** Relative expression of γ-H2AX was analyzed by western blot in HUVECs and HK-2 cells with ACTB as a loading control.

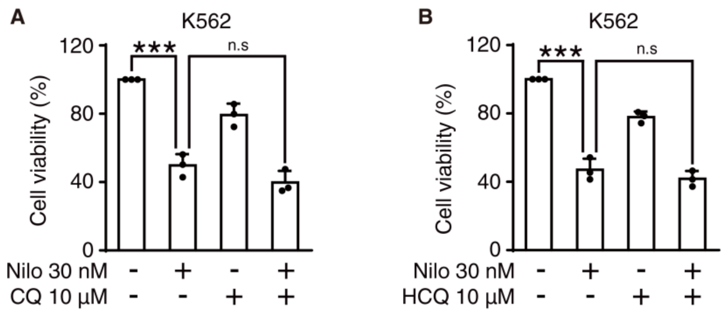

Supplemental Figure 15. CQ or HCQ's effect on the anti-cancer activity of nilotinib. (A and B) K562 cells were treated with 30 nM nilotinib or/and 10 μM CQ or

HCQ for 24 h. The cell viability was analyzed by CCK-8 assay. n = 3 independent experiments. The results are presented as the mean ± SD. The P value was calculated by one-way ANOVA (Dunnett's multiple comparisons test). n.s = no significance; ***P < 0.001.

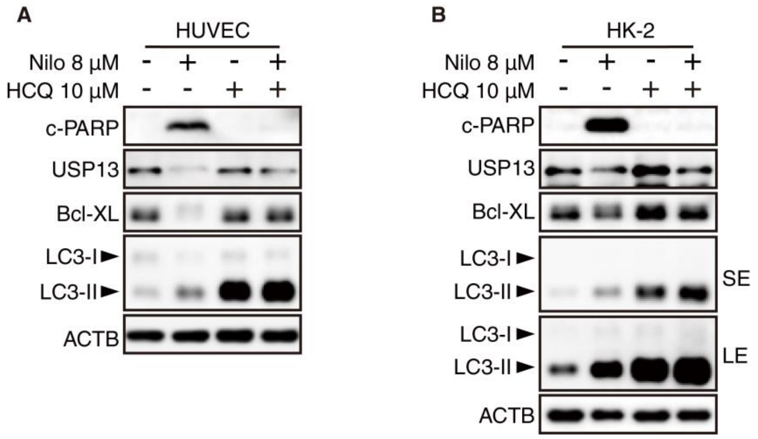

Supplemental Figure 16. HCQ alleviates cell apoptosis in kidney cells via the regulation of USP13 and Bcl-XL. (A and B) HUVECs and HK-2 cells were treated with 8 μM nilotinib or/and 10 μM HCQ for 24 h. The expression levels of c-PARP, USP13, Bcl-XL and LC3 were determined by western blot with ACTB as a loading control.

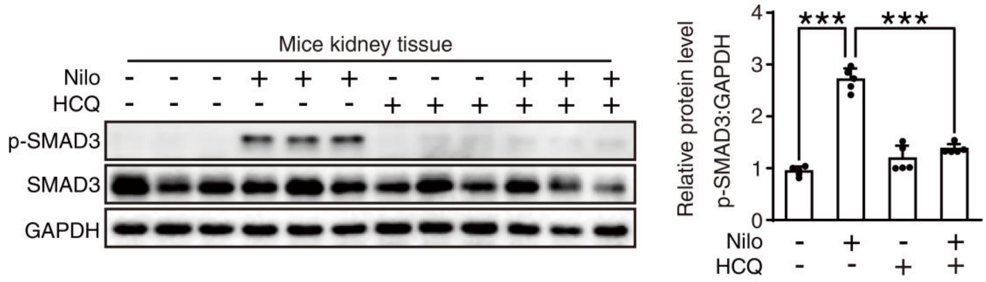

Supplemental Figure 17. HCQ rescued nilotinib-induced elevation of phosphorylated SMAD3. Relative expression of p-SMAD3 and SMAD3 in kidney tissues from control, nilotinib-, HCQ-, nilotinib plus HCQ-treated group (n = 5 per group) were analyzed by western blot. GAPDH was used as a loading control. The results are presented as the mean ± SD. The P value was calculated by one-way ANOVA (Dunnett's multiple comparisons test). ***P < 0.001.

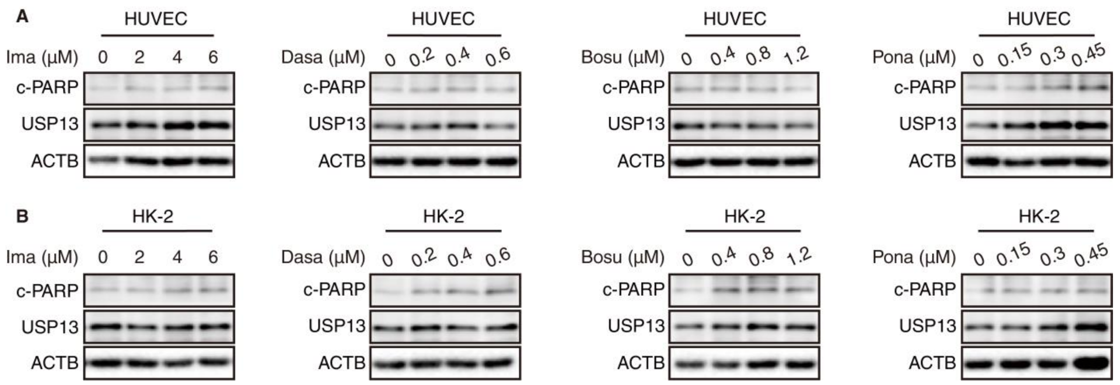

Supplemental Figure 18. BCR-ABL1 inhibitors' effect on the expression of USP13. (A) HUVECs and **(B)** HK-2 cells were treated with 0, 1, 2, 3 times maximum plasma concentration of imatinib, dasatinib, bosutinib and ponatinib for 24 h. The protein level of c-PARP and USP13 were analyzed by western blot with ACTB as a loading control.
